# Integration of Non-Targeted Metabolomics and Targeted Quantitative Analysis to Elucidate the Synergistic Antidepressant Effect of Bupleurum Chinense DC*-*Paeonia Lactiflora Pall Herb Pair by Regulating Purine Metabolism

**DOI:** 10.3389/fphar.2022.900459

**Published:** 2022-06-30

**Authors:** Jiajun Chen, Tian Li, Xuemei Qin, Guanhua Du, Yuzhi Zhou

**Affiliations:** ^1^ Modern Research Center for Traditional Chinese Medicine, Shanxi University, Taiyuan, China; ^2^ The Key Laboratory of Chemical Biology and Molecular Engineering of Ministry of Education, Shanxi University, Taiyuan, China; ^3^ The Key Laboratory of Effective Substances Research and Utilization in TCM of Shanxi Province, Taiyuan, China; ^4^ Institute of Materia Medica, Chinese Academy of Medical Sciences and Peking Union Medical College, Beijing, China

**Keywords:** *Bupleurum chinense* DC-*Paeonia lactiflora* Pall, compatibility mechanism, depression, cortex, xanthine, oxidative stress, inflammation

## Abstract

*Bupleurum chinense* DC (Chaihu)*-Paeonia lactiflora* Pall (Baishao) is among the most accepted herb pairs in many classic antidepressant prescriptions. Our previous study has shown that the Chaihu–Baishao herb pair (CBHP) had a better antidepressant effect than Chaihu or Baishao. Nevertheless, the synergistic antidepressant mechanism of this herb pair was not clearly understood. This study aimed to investigate the compatibility mechanism of Chaihu and Baishao for treating depression through a strategy of non-targeted metabolomics combined with targeted quantitative analysis and molecular biology techniques. First, the compatibility effects of CBHP were assessed by the chronic unpredictable mild stress (CUMS) rat model. Next, cortex metabolomics based on ultra-high-performance liquid chromatography combined with quadrupole orbitrap mass spectrometry (UPLC-Q-Orbitrap/MS) was used to discover the metabolic pathway that was synergistically regulated by CBHP. Based on the results of metabolomics analysis, metabolites were quantitatively validated by UPLC-MS/MS combined with the MRM mode in the crucial metabolic pathway. In addition, the signaling pathway associated with this metabolic pathway was detected by molecular biology techniques to further identify the biological meaning of the crucial metabolite on the synergistic antidepressant effect of CBHP. The antidepressant effect of CBHP was significantly better than that of Chaihu or Baishao single administrated in the behavioral test. According to cortex metabolomics, a total of 21 differential metabolites were screened out, and purine metabolism was selected as the crucial metabolic pathway by the enrichment analysis of differential metabolites. Subsequently, purine metabolism was confirmed as disorder in the CUMS group by targeted quantitative analysis, CBHP regulated more purine metabolites (six) than individual administration (two and two). The results showed that purine metabolism was modulated by CBHP through synergistically decreasing xanthine levels and inhibiting the conversion of xanthine dehydrogenase (XDH) to xanthine oxidase (XOD). Finally, the synergistic regulation effect of CBHP on xanthine synthesis was found to be related to inhibition of malondialdehyde (MDA) production, Nod-like receptor protein 3 (NLRP3) inflammasome expression, and interleukin (IL)-1*β*, IL-6, and tumor necrosis factor (TNF)-*α* secretion. The present study demonstrated that the regulation of purine metabolism, the suppression of oxidative stress, and inflammatory responses in the cortex were involved in the synergistic antidepressant effect of CBHP.

## Graphical Abstract

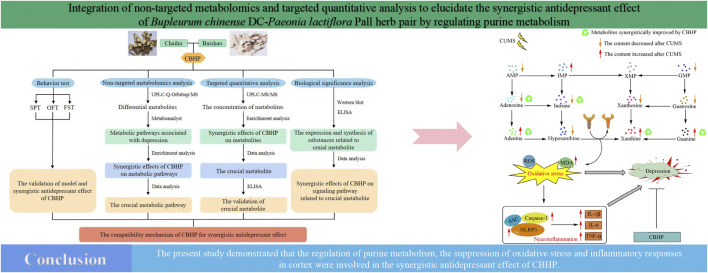

## 1 Introduction

Depression is a widespread mental disorder that seriously disturbs human life (Xinyu Zhou et al., 2019). With the development of society, the increase of pressure and lack of emotion make the incidence of depression increase year by year. However, there is a lack of antidepressant drugs that have significant effects and few side effects (Hughes et al., 2017; Rothmore, 2020; Wu et al., 2020; Kendrick, 2021). Therefore, antidepressant drugs with multi-targets, safety, and efficiency need to be developed urgently.

Traditional Chinese medicine (TCM) is widely used in the treatment of depression because of its advantages of multiple targets and low side effects. Herbal compatibility, as the basic characteristic of TCM, is of great significance to the research of TCM modernization (Liu et al., 2020). Chaihu and Baishao were used together in many classic antidepressant prescriptions, such as Chaihushugan san, Sini san, and Xiaoyao san (Jia et al., 2018; Zhou et al., 2018; Zhu et al., 2019). Our previous study has shown that Chaihu and Baishao had antidepressant effects, but CBHP had a better antidepressant effect (Li et al., 2021). Subsequently, the synergistic antidepressant effect of CBHP was found to be related to peripheral metabolic regulation (Li et al., 2021), whereas the effect of Chaihu, Baishao, and CBHP on the brain (closely related to the pathogenesis of depression) was still unclear.

Currently, the cortex was thought to be the main part for regulating mood disorders (Lei et al., 2020; Rupprechter et al., 2020). Therefore, the cortex would be used as the research object to dissect the compatibility mechanism of Chaihu and Baishao in this study. Metabolomics was a promising tool to research the compatibility mechanism of TCM or combinatorial drugs (Cui et al., 2017). We previously used non-targeted metabolomics to study the effect of Chaihu, Baishao, and CBHP on peripheral metabolic pathways (Li et al., 2021). Nevertheless, non-targeted metabolomics cannot accurately reflect the changes in metabolite content. For that reason, an efficient strategy of non-targeted metabolomics combined with MRM-targeted quantitative analysis was adopted in this study (Chen L. et al., 2020; Zheng et al., 2020). Substantial metabolite information was obtained from non-targeted metabolomics to ensure the high coverage rate of the metabolites, and metabolite content was quantified by MRM-targeted quantitative analysis to further guarantee the high quality of data.

Numerous studies have found that external stress led to inflammatory reactions in the rat cortex, inducing the formation of the NLRP3 inflammasome and the release of inflammatory factors, such as IL-6, IL-1*β*, and TNF-*α* (Geng et al., 2019; Cai et al., 2020; Xie et al., 2020). In a previous study, inflammation was considered to be the important cause of depression (Beurel et al., 2020), while the disturbance of purine metabolism was thought to be closely related to depression induced by inflammation (Wu et al., 2017). Inflammation could be induced by disordered purine metabolism (Linden et al., 2019), whereas purine metabolites that played a key regulatory role were not well-understood. Our previous study showed that the combination of Chaihu and Baishao could promote the anti-inflammatory activity of Chaihu or Baishao (Chen et al., 2021). However, it was unclear whether CBHP could promote an anti-inflammatory effect through the regulation of purine metabolism and further produce a synergistic antidepressant effect.

**FIGURE 1:**
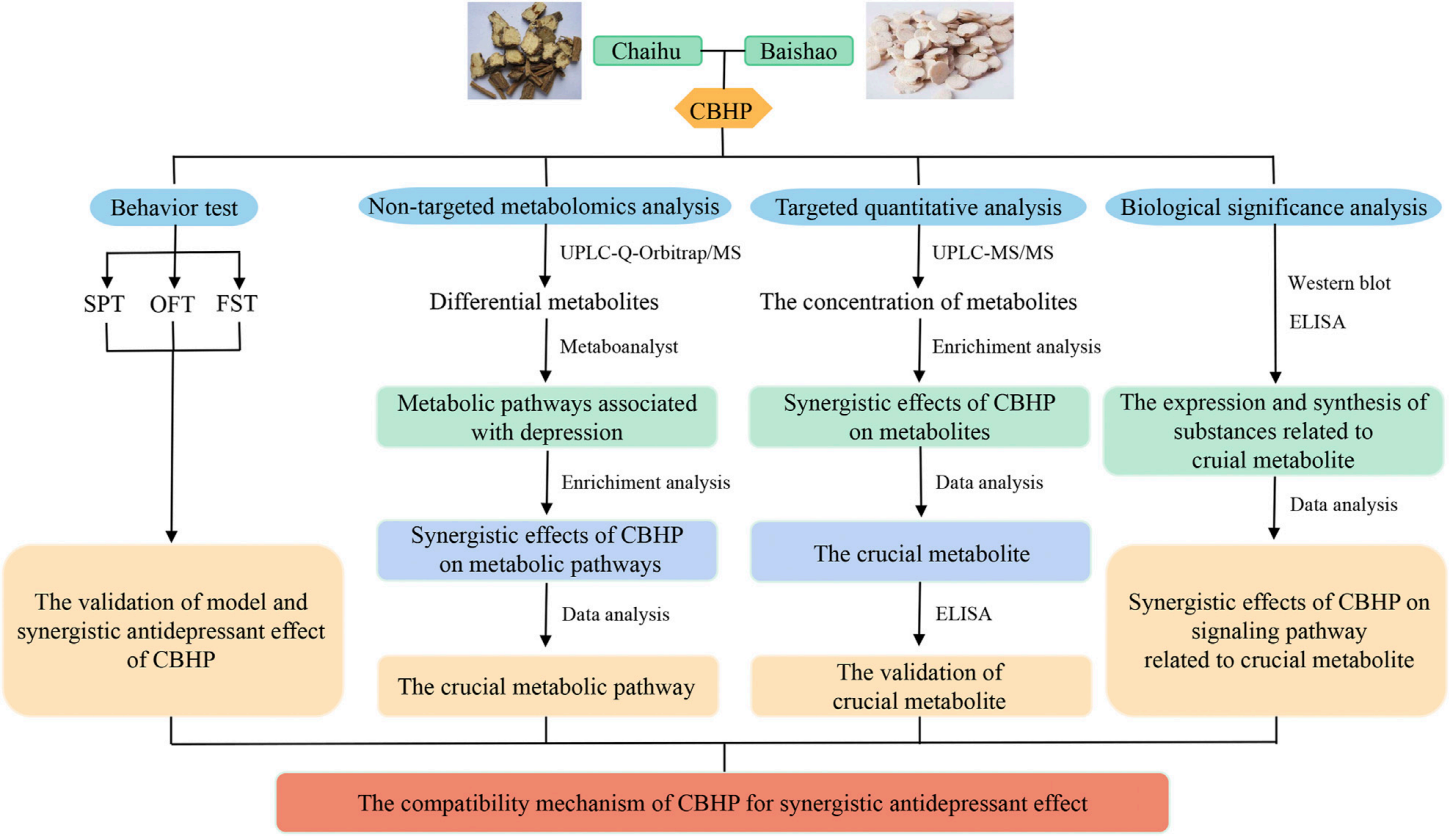
Workflow for studying the antidepressant compatibility mechanism of Chaihu and Baishao.

In the present study, purine metabolism was screened as the key pathway regulated synergistically by Chaihu and Baishao through cortex non-targeted metabolomics. Next, the related differential metabolites were verified by MRM-targeted quantitative analysis. Subsequently, western blots and enzyme-linked immunosorbent assay (ELISA) kits were used to elucidate the potential biological role of crucial metabolic pathways (Figure 1). Briefly speaking, this study provided a new idea for the compatibility mechanism of Chaihu and Baishao for treating depression.

## 2 Materials and Methods

### 2.1 Chemicals and Reagents

Chaihu (batch number: 180801) and Baishao (batch number: 181102) were both acquired from Shanxi Renhetang Co., Ltd. and authenticated by Professor Xuemei Qin in the Pharmacognosy Department of Shanxi University. Voucher specimens were deposited in the Modern Research Center for Traditional Chinese Medicine of Shanxi University, labeled as YZ-2018-0403002 (Chaihu) and YZ-2018-0403001 (Baishao). Methanol, acetonitrile, and formic acid (HPLC-grade) were obtained from Thermo Fisher Scientific Inc. Deionized water was purified by using a Milli-Q system. Sodium hydroxide was purchased from Tianjin Zhiyuan Chemical Reagent Co., Ltd. Phosphate-buffered saline (PBS) was obtained from Sangon Biotech Co., Ltd. In addition, the reference substances for targeted quantitative analysis were collected from Target Molecule Corp.

### 2.2 Preparation of Herbs Extracts

Two kilograms of Chaihu, Baishao, and CBHP (1:1, w/w) were immersed in 70% ethanol (1:8, w/v) for 2 h. Subsequently, the extracting solutions were concentrated in vacuum and lyophilized into powder and stored at −80°C (the drug extraction ratios of Chaihu, Baishao, and CBHP were 19.4%, 14.8%, and 15.6%, respectively).

### 2.3 Animals and Drug Administration

**FIGURE 2:**
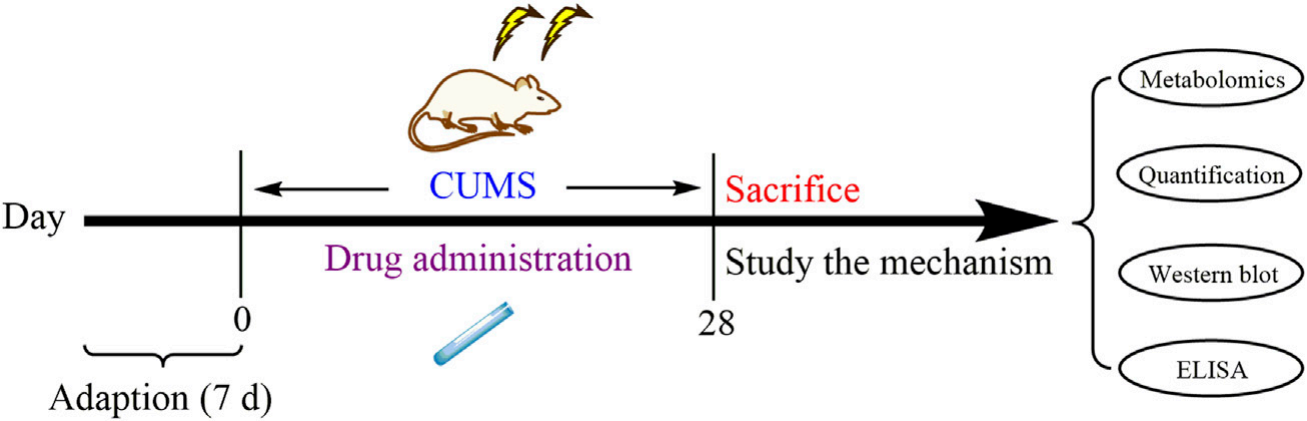
Experimental procedure design.

All animal experiments were performed under the NIH Guidelines for Care and Use of Laboratory Animals (United States) and the Prevention of Cruelty to Animals Act (1986) of China, and the experiments had also obtained approval from the Animals Ethics Committee of Shanxi University (approval number SXULL2019018). All male Sprague–Dawley rats (180 ± 20 g) were commercially obtained from Beijing Weitong Lihua Experimental Animal Technology Co. Ltd. (license No. SCXK 2016–0011). All rats were maintained under standard laboratory conditions (temperature of 20–24°C and relative humidity of 45%–55%) with a 12/12 light–dark cycle (lighting at 8 o’clock and turning off the lights at 20 o’clock) and free access to filtered water and standard food. After 1 week acclimatization, according to the preliminary behavior test, 81 rats with similar indicators were randomly divided into nine groups: control group, model group (CUMS), positive drug group (V, venlafaxine, 35 mg/kg), low-dose Chaihu group (CL, 7.5 g crude drug/kg), low-dose Baishao group (BL, 7.5 g crude drug/kg), low-dose CBHP group (CBL, 7.5 g crude drug/kg), high-dose Chaihu group (CH, 15 g crude drug/kg), high-dose Baishao group (BH, 15 g crude drug/kg), and high-dose CBHP group (CBH, 15 g crude drug/kg). Regarding the dosage setting, we based it on the proportion of Chaihu and Baishao in Xiaoyao San prescription. The antidepressant effects of Xiaoyao San have been studied in our group, and we have previously determined that the best dose of Xiaoyao San in CUMS rats was 46.2 g crude drug/kg (Chen C. et al., 2020); therefore, 7.5 g crude drug/kg was set as the low dose of Chaihu, Baishao, and CBHP, and its double dose was set as the high dose. Drugs were administered by oral gavage daily for 28 days, and the dosing volume depended on the body weight of the rats (10 ml/kg). Compared with the administration groups, the control group and CUMS group were given distilled water by oral gavage for 28 days. The CUMS procedure was referred to the previous study (Gong et al., 2019), and a random stimulus was given to individually raised rats from 9 to 11 a.m. every day, including swimming in the water at 4°C for 5 min, unpredictable foot shocks for 2 min (36V, one shock/2 s, 10 s duration), noises for 3 h (60 dB), exposure to a hot room at 45°C for 10 min, food deprivation for 24 h, water deprivation for 24 h, constraint for 2 h, day–night reversal (12 h/12 h), and tail clamp for 2 min. The experimental design could be seen in Figure 2.

### 2.4 Behavior Test

#### 2.4.1 Body Weight

The rats were weighed at 0, 1, 2, 3, and 4 weeks of the experiment.

#### 2.4.2 Open-Field Test

The open-field apparatus was a black box with a bottom (100 × 100 × 40 cm) divided into 25 identical squares. First, each rat stayed in the center square at the bottom of the open-field apparatus for 1 min to adapt to the environment. Next, the number of crossings and rearing was recorded within 4 min. In order to eliminate the interference caused by animal odor, the equipment was cleaned with ethanol solution during the experiment.

#### 2.4.3 Sucrose Preference Test

The sucrose preference test was performed on 0 and 4 weeks of CUMS. Before the experiment, the rats were individually housed and provided two bottles of 1% sucrose water for adaption to sucrose solution. On the first day, two bottles of 1% sucrose water were placed, and one of them was changed to water 1 day later. After the abovementioned sucrose preference rate training, all animals were deprived of food and water for 12 h, and then the rats had *ad libitum* access to sucrose water or water for another 12 h. The sucrose preference rate of rats was calculated according to the consumption of the two drinks: sucrose preference rate (%) = sucrose water intake/(sucrose water intake + daily water intake) × 100%.

#### 2.4.4 Forced Swimming Test

The forced swimming test was performed by using a cylindrical water container (25 ± 1°C, depth of 30 cm). On the day before FST, each rat received 15 min of forced swimming training. FST was performed on the following day, each rat was adapted to 2 min, and the immobility time of the last 4 min was observed. It was recorded as the rat immobility time: did not show a struggle, kept the head above the water surface, and avoided sinking or floating.

### 2.5 Metabolomics Analysis

#### 2.5.1 Sample Preparation

Twenty mg of the cerebral cortex sample was homogenized for 60 s in 400 μl chilled acetonitrile-water (3:1, v/v), blended for 1 min, and centrifuged at 4°C and 12500 rpm for 10 min to precipitate the proteins to obtain a protein-free supernatant. The supernatant was collected and blow-dried under nitrogen. The dried residue was reconstituted in 100 μl of acetonitrile water (1:1, v/v), centrifuged at 4°C/12500 rpm for 10 min, and an aliquot of 5 μl was injected for UPLC-Q-Orbitrap/MS analysis. In addition, 10 μl from each test sample was thoroughly mixed to use as a quality control (QC) sample.

#### 2.5.2 UPLC-Q-Orbitrap/MS Method

Thermo-Fisher Dionex UltiMate 3000 UPLC-Q Exactive Orbitrap-MS and Xcalibur workstation were used to obtain LC-MS raw data. For the chromatographic separation of the cerebral cortex, the sample was analyzed on an ACQUITY UPLC HSS T3 column. The column temperature was maintained at 35°C, the flow rate was set at 0.2 ml/min, and the injection volume was 5 μl; the mobile phase and gradient are shown in Supplementary Table S1. Mass spectrometry conditions: samples were analyzed under positive and negative ionization modes *via* a heated electrospray ionization (HESI) source; the scan mode was “Full Scan 35000 FWHM/dd-MS2” (Resolution 17500, NCE 25, Stepped NCE 50%); the spray voltage was set to 3.6 kV for the positive mode and 2.5 kV for the negative mode; capillary temperature, 320°C; sheath gas flow, 35 arbitrary units; aux gas flow, 10 arbitrary units; scan range, *m*/*z* 50–1500.

#### 2.5.3 Data Analysis

The raw data were imported to “Compound Discoverer 2.0” (Thermo Fisher, United States) to obtain the matched peak data, and the peak area data were normalized in Microsoft Excel (2016). Furthermore, SIMCA-P software (version 14.1, Umetrics, Sweden) was used for the principal components analysis (PCA), partial least-squares discriminant analysis (PLS-DA), and orthogonal partial least-squares (OPLS) analysis of the data from both positive and negative modes. The acquired normalized data from LC-MS were imported into SIMCA-P to perform multivariate data analysis. The altered metabolites were selected between the control group and CUMS group based on VIP values (VIP >1) and *t*-test (*p* < 0.05). Next, the screened metabolites were identified by comparison with the *m*/*z*, retention time, formula, and the MS/MS fragmentation information in the Human Metabolome Database (http://www.hmdb.ca/), Pubchem (https://pubchem.ncbi.nlm.nih.gov/) and Massbank (http://www.massbank.eu/). Finally, MetaboAnalyst (https://www.metaboanalyst.ca/) was used for metabolic pathway analysis and enrichment analysis.

### 2.6 Quantitative Analysis of Purine Metabolites

#### 2.6.1 Treatment of the Reference Substances

Each standard was individually prepared in the 0.01 mol/L NaOH because of the low solubility in neutral solutions. The hybrid standard liquid was prepared by constant PBS dilution for standard curve fabrication and methodology investigation. The internal standard (IS) solution was prepared by mixing IS1 and IS2 with PBS to a final concentration of 1.2 μg/ml for IS1 (2-chloroadenosine) and 0.15 μg/ml for IS2 (Galanthamine). Concentrations of reference substances are shown in the Supplementary Table S2.

#### 2.6.2 Methodological Investigation

QC samples of high (close to 75% of the upper limit of the standard curve), medium (close to the concentrations in samples), and low [within three times of the lower limit of quantification (LLOD)] concentration were prepared by the mixed standard solution of different concentrations, as shown in Supplementary Table S3. Selectivity was used to verify whether the analytical method could distinguish metabolites of purine metabolism, internal standards, and other components in the sample. Subsequently, chromatogram differences between blank solution, QC2 sample, and normal cerebral cortex sample were compared. The linearity relationship was analyzed in at least six concentrations of reference substance and its detected peak area. Inter-day and intra-day precision were obtained by measuring LLOQ, QC1, QC2, and QC3 solution. The stability of QC1 and QC3 solutions under repeated freeze–thaw three times at −20°C and placed in an auto sampler at 4°C for 24 h had been investigated. In addition, the method validation also included the matrix effect, extraction recovery rate, and dilution effect. The relative standard deviation (RSD) value of each item is not more than 15%, and the RSD value of LLOQ is not more than 20%.

#### 2.6.3 Samples Preparation

Twenty mg of the cerebral cortex sample was mixed with 20 μl of IS solution and 400 μl acetonitrile-water (3:1, v/v), blended for 1 min, and centrifuged at 4°C and 12500 rpm for 10 min to precipitate the proteins. The supernatant was collected and blow-dried under nitrogen. The dried residue was reconstituted in the 100 μl acetonitrile-water (1:1, v/v), centrifuged at 4°C/12500 rpm for 10 min, and an aliquot of 5 μl was injected into the UPLC-MS/MS system for assay.

#### 2.6.4 UPLC-MS/MS Analysis

Chromatographic separation was conducted on an Agilent 1290 series UPLC system (Agilent Technologies, CA, United States) with an ACQUITY UPLC HSS T3 column (Waters, United States, 2.1 mm × 100 mm, 1.8 μm). The optimum separation was obtained under gradient elution with phase A (0.1% v/v formic acid in water) and phase B (acetonitrile). The gradient elution was carried out as follows: 0–2 min, 0% B; 2–5 min, 0–1% B; 5–6 min, 1–3% B; 6–6.5 min, 3–10% B; 6.5–8 min, 10–20% B; 8–9 min, 20–35% B; 9–11 min, 35–95% B; 11–12 min, 95% B; 12–14 min, 95–0% B; 15 min, 0% B. The flow rate was 0.1 ml/min, the injected volume was 5 μl, and the column temperature was maintained at 35°C. Mass spectrometric detection was performed using an AB SCIEX API 3200MD equipped with an electron spray ionization (ESI) source. The parameters in the source were set as follows: drying gas temperature, 300°C; gas flow rate, 12 L/min; sheath gas temperature, 350°C; sheath gas flow rate, 12 L/min; nebulizer, 45 psig; capillary voltage, 5500 V; nozzle voltage, 500 V. Nitrogen was used as the drying and collision gas. Detection was conducted in the positive mode using multiple reaction monitoring (MRM).

### 2.7 Measurement of Xanthine, XOD, XDH, and MDA

The level of MDA was determined by a commercial assay kit (Nanjing Jiancheng Bioengineering Institute, China). The content of xanthine and the activities of XOD and XDH were quantified by using commercially available ELISA kits (AndyGene Biotechnology Co., Ltd., China).

### 2.8 Western Blot

The cerebral cortex samples (*n* = 5) were homogenized in RIPA lysis buffer containing 1% PMSF and incubated on ice for 60–80 min. The homogenates were centrifuged at 12,000 rpm for 20 min at 4°C. Next, the supernatants were collected. The concentration of total protein was determined by BCA protein assay kits. The proteins were separated by using 6% or 10% SDS-PAGE gels and transferred to PVDF membranes after blocking with Tris-buffered saline solution (TBST) containing 5% (w/v) non-fat milk for 2 h and incubating with the appropriate primary antibodies at 4°C overnight: NLRP3 (1:1000), caspase-1 (1:1000), IL-1*β* (1:1000), *β*-actin (1:1000). The membranes were washed three times by TBST and incubated with HRP-conjugated antibodies (1:5000) at room temperature for 2 h. After three washes in TBST, the membranes were scanned using the fluorescent scanner.

### 2.9 Measurement of IL-1*β*, IL-6, and TNF-*α*

The levels of IL-1*β*, IL-6, and TNF-*α* were quantified by using commercially available ELISA kits (Shanghai Xitang Biological Technology Co., Ltd., China).

### 2.10 Statistical Analysis

The results of the experiment were expressed as the means ± SD and analyzed by GraphPad Prism 9. The statistical difference between the two groups was compared by *t*-test (*p <* 0.05) and the significant difference between more groups was compared by one-way ANOVA.

## 3 Results

### 3.1 Comparison of Antidepressant Effect Between Chaihu and Baishao Before and After Compatibility

After 4 weeks of stress stimulation, the rats showed significant depressive-like behavior. Among them, body weight (Supplementary Figure S1A, *p* < 0.001), sugar water preference rate (Supplementary Figure S1B, *p* < 0.001), crossing numbers (Supplementary Figure S1C, *p* < 0.001), and rearing scores (Supplementary Figure S1D, *p* < 0.001) of CUMS rats decreased significantly, and the immobility time (Supplementary Figure S1E, *p* < 0.001) increased significantly in the FST. These results indicated that the depressed model was successfully established. With the treatment of drugs, the depressive-like behavior of CUMS rat was significantly improved, and the effect of CBHP was better than that of single herbs, close to the positive drug. The results showed that the antidepressant effects of drugs were better reflected in the low-dose groups, so 7.5 g crude drug/kg of drugs was used to investigate the compatibility mechanism of Chaihu and Baishao in subsequent studies. Critically, these results were consistent with those of a previous report (Li et al., 2021).

### 3.2 Metabolites and Metabolic Pathway Analysis Based on Cortex Nontargeted Metabolomics

#### 3.2.1 Examination of Instrument Stability

The typically based peak intensity chromatograms of cerebral cortex samples were analyzed in both positive and negative modes (Supplementary Figure S2). In addition, during the batch injection, a QC sample was inserted after every five samples for instrument stability monitoring. To ensure the reliability of the subsequent multivariate analysis, we examined the instrument stability through the RSD values of the relative peak areas of 20 random ions in the six QC samples and the deviation of QC samples in the 1D PCA. The results showed that the RSD values of the relative peak areas of 20 random ions ranged from 4.14% to 14.15% (Supplementary Table S4), and the deviation of six QC samples was within 2SD (Supplementary Figure S3). Thus, the instrument was stable and met the requirement for subsequent multivariate analysis.

#### 3.2.2 Multivariate Data Analysis

**FIGURE 3:**
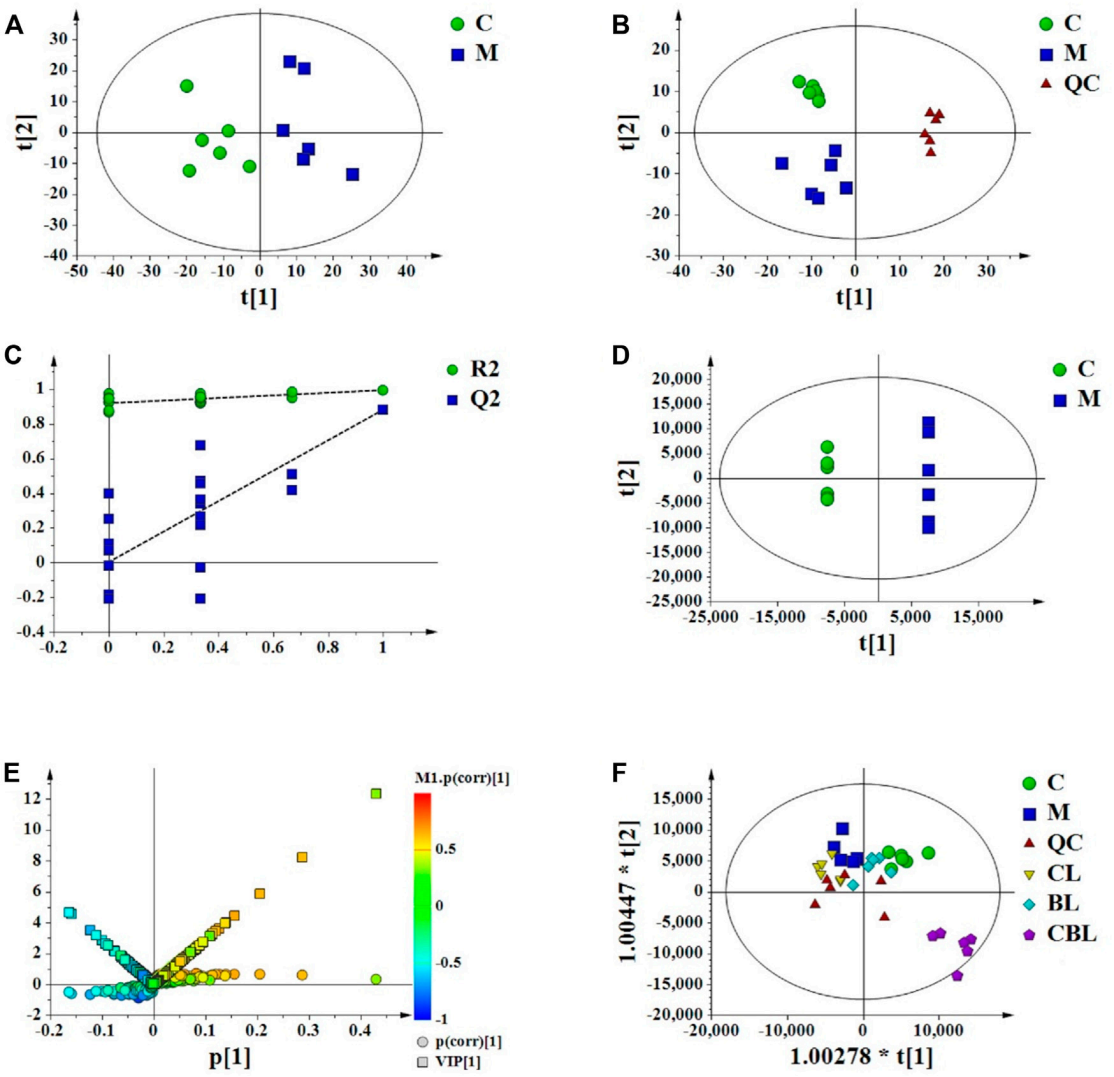
Multivariate data analysis from UPLC-MS/MS. **(A)** PCA scores of the control **(C)** and CUMS (M) groups; **(B)** PCA scores of the control, CUMS, and QC groups; **(C)** PLS-DA model validation diagram; **(D)** PLS-DA scores of the control and CUMS groups; **(E)** V-S-plot; **(F)** OPLS-DA score plots of control **(C)**, CUMS, Chaihu (CL), Baishao (BL), and CBHP (CBL) groups.

The PCA score plots indicated that the CUMS group was separated from the control group (Figure 3A) and the instrument was stable (Figure 3B). The permutation test was used to validate the PLS-DA model (Figure 3C). In the PLS-DA model, R^2^X, R^2^Y, and Q^2^ in UPLC-Q-Orbitrap/MS were 0.615, 0.995, and 0.891, respectively, which suggested that the model of PLS-DA was reliable. The score plots of PLS-DA also showed that the control group was significantly separated from the CUMS group (Figure 3D), and the result was consistent with that of PCA score plots. The variable of VIP >1 in scatter plots (Figure 3E) combined with a *t*-test (*p* < 0.05) was used to determine the related metabolites of depression. In addition, the distribution of five groups in OPLS-DA score plots (Figure 3F) showed that the compatibility group could be significantly separated from the single-herb groups and CUMS group.

#### 3.2.3 Analysis of Endogenous Metabolites

**FIGURE 4:**
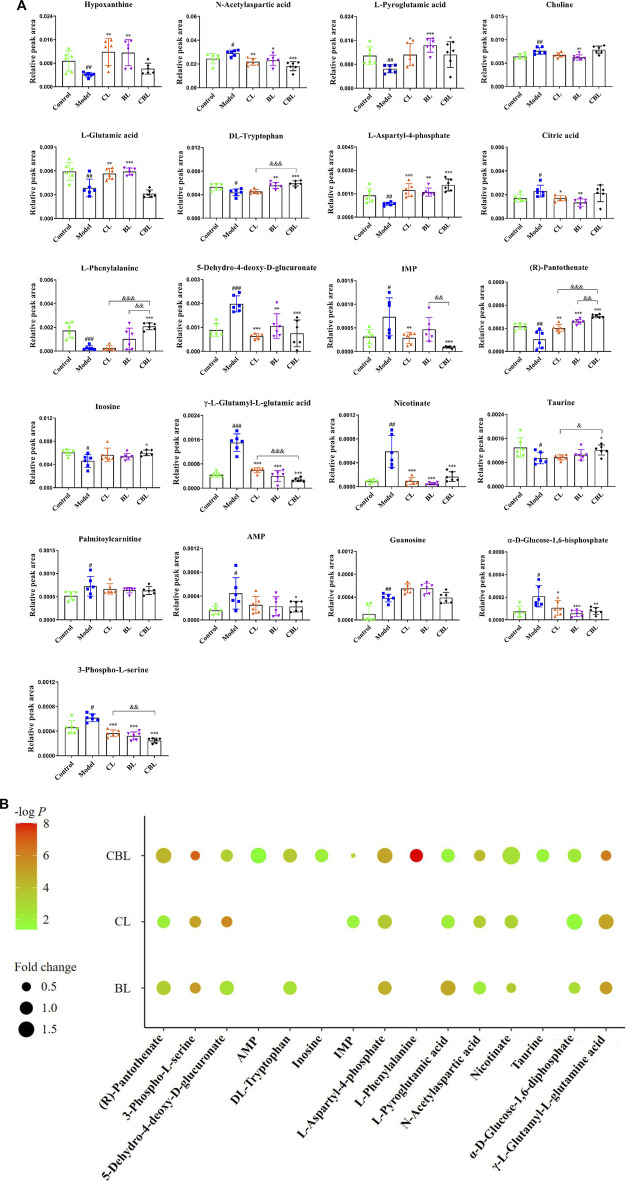
**(A)** Comparison of relative peak areas of differential metabolites in the rat cortex of each group. $\bar{x}$ ± s, *n* = 6. Compared with the control group: ^#^ *p* < 0.05, ^##^ *p* < 0.01, and ^###^ *p* < 0.001; compared with the CUMS (Model) group: ^*^ *p* < 0.05, ^**^ *p* < 0.01, and ^***^ *p* < 0.001; compared with the CBHP (CBL) group: ^&^ *p* < 0.05, ^&&^ *p* < 0.01, and ^&&&^ *p* < 0.001. **(B)** Enrichment analysis of differential metabolites. -log *P*: the significance of different groups compared with the CUMS group; Fold change: relative to the mean of control group.

A total of 22 differential metabolites related to depression were screened out by multivariate data analysis (Table 1). Among them, triethyl phosphate (PubChem ID: 87574509) was identified as an exogenous substance, so this substance was excluded from endogenous metabolites. The peak area comparison between various groups based on different metabolites is shown in Figure 4A. Compared with the control group, 12 differential metabolites were significantly increased in the CUMS group, including N-acetylaspartic acid; choline; citric acid; 5-dehydro-4-deoxy-D-glucuronate; inosine monophosphate (IMP); γ-glutamylglutamic acid; nicotinate; palmitoylcarnitine; adenosine monophosphate (AMP); guanosine; and α-D-Glucose-1,6-bisphosphate and 3-Phospho-L-serine. In addition, nine differential metabolites were significantly decreased in the CUMS group, namely: inosine; hypoxanthine; L-pyroglutamic acid; L-glutamic acid; DL-tryptophan; L-phenylalanine; L-aspartyl-4-phosphate; taurine, and (R)-pantothenate. Among them, 13 metabolites were regulated by the Chaihu group, 14 metabolites were regulated by the Baishao group, and 15 metabolites were regulated by the CBHP group. Among the 15 metabolites, nine metabolites were simultaneously regulated by Chaihu and Baishao, and four metabolites were only regulated by CBHP (Figure 4B).

**TABLE 1:** Differential metabolites associated with depression in the rat cortex detected by UPLC-Q-Orbitrap/MS.

	Metabolites	Formula	TR (min)	Scan mode	m/z	*p* value	VIP	HMDB	Trend
1	Hypoxanthine	C_5_H_4_N_4_O	1.28	+	137.05	0.0087	7.3	0000157	↓
2	N-acetylaspartic acid	C_6_H_9_NO_5_	1.27	+	176.06	0.0414	4.5	0000812	↑
3	L-pyroglutamic acid	C_5_H_7_NO_3_	1.15	+	130.05	0.0073	6.7	0000267	↓
4	Choline	C_5_H_13_NO	1.01	+	104.11	0.0015	2.5	0000097	↑
5	L-glutamic acid	C_5_H_9_NO_4_	1.19	+	148.06	0.0195	3.7	0000148	↓
6	DL-tryptophan	C_11_H_12_N_2_O_2_	4.90	+	188.07	0.0144	2.9	0013609	↓
7	Citric acid	C_6_H_8_O_7_	1.24	−	191.02	0.0214	2.2	0000094	↑
8	L-phenylalanine	C_9_H_11_NO_2_	1.81	+	166.09	0.0028	5.1	0000159	↓
9	L-aspartyl-4-phosphate	C_4_H_8_NO_7_P	1.25	+	214.01	0.0318	2.7	0012250	↓
10	5-dehydro-4-deoxy-6-D-glucuronate	C_6_H_8_O_6_	1.42	−	175.02	0.0382	2.8	METPA0464	↑
11	IMP	C_10_H_13_N_4_O_8_P	1.49	+	349.05	0.0171	1.8	0000175	↑
12	Inosine^a^	C_10_H_12_N_4_O_5_	1.39	+	269.09	0.0014	3.2	0000195	↓
13	γ-L-glutamyl-L-glutamic acid	C_10_H_16_N_2_O_7_	1.35	+	277.10	0.0001	2.1	0011737	↑
14	Nicotinate	C_6_H_5_NO_2_	1.21	+	124.04	0.0068	2.2	0001488	↑
15	Taurine	C_2_H_7_NO_3_S	1.01	−	124.01	0.0114	1.4	0000251	↓
16	Palmitoylcarnitine	C_23_H_45_NO_4_	11.87	+	400.34	0.0338	1.1	0000222	↑
17	AMP^a^	C_10_H_14_N_5_O_7_P	1.23	+	348.07	0.0118	1.6	0000045	↑
18	Guanosine^a^	C_10_H_13_N_5_O_5_	3.11	+	284.10	0.0137	1.8	0000133	↑
19	α-D-glucose-1,6-bisphosphate	C_6_H_14_O_12_P_2_	1.34	−	338.99	0.0172	1.3	0003514	↑
20	(R)-Pantothenate	C_9_H_17_NO_5_	4.71	+	220.12	0.0024	1.5	0000210	↓
21	3-phospho-L-serine	C_3_H_8_NO_6_P	1.02	+	186.02	0.0036	1.1	0001721	↑
22	Triethyl phosphate	C_6_H_15_O_4_P	6.17	+	183.08	0.0182	1.2	—	↓

“↑” or “↓” means the metabolite significantly increased or decreased in the CUMS, group compared with the control group.

a Validated with the standard.

#### 3.2.4 Pathway Analysis

**FIGURE 5:**
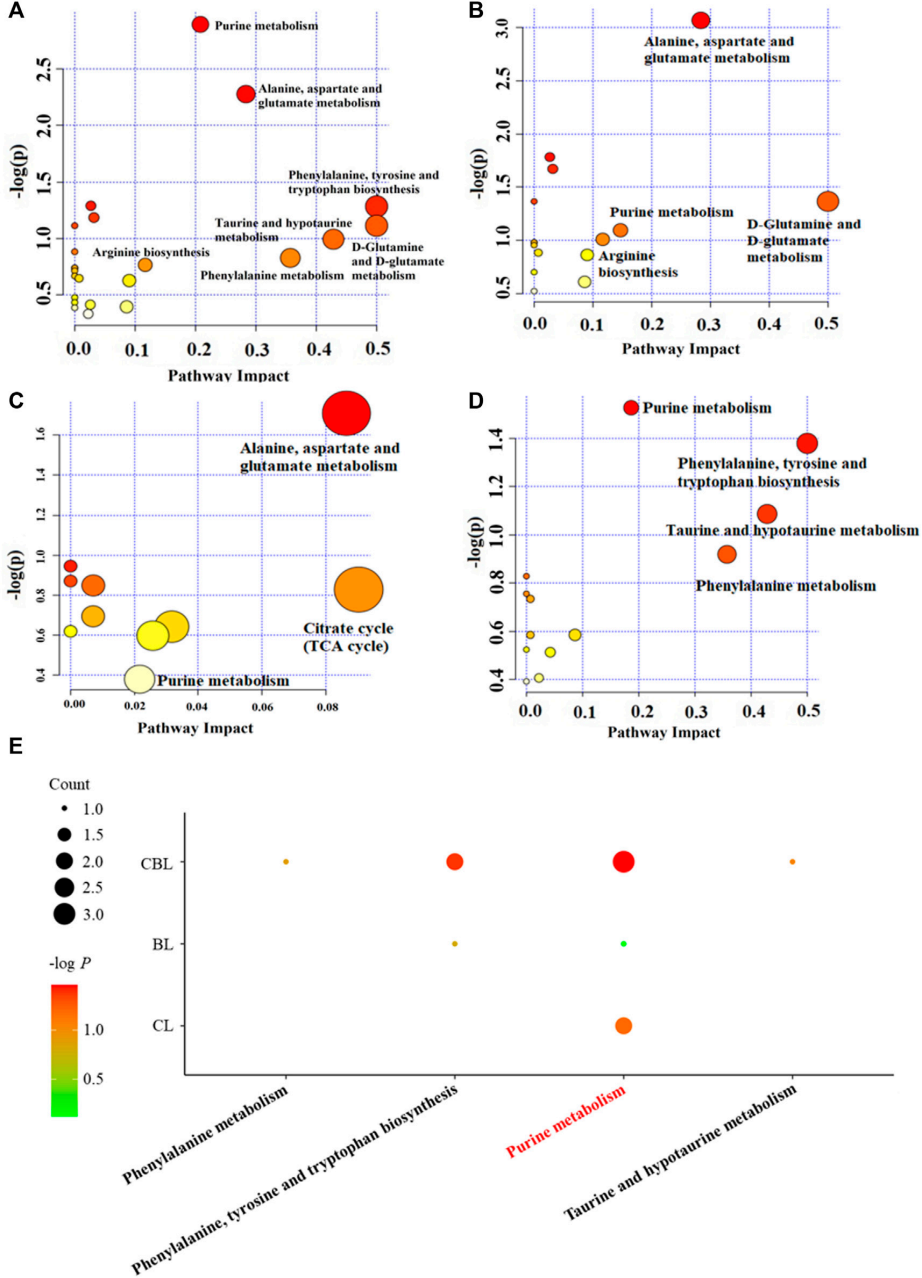
Pathway analysis of the cortex in differently treated rats. **(A)** CUMS group, **(B)** Chaihu group, **(C)** Baishao group, **(D)** CBHP group, and **(E)** enrichment analysis of differential metabolic pathways. Count: the number of metabolites that were regulated in the corresponding pathway; -log *P*: the significance of corresponding metabolic pathways.

Depression-related metabolites in the trial were imported into the “MetaboAnalyst” online database (https://www.metaboanalyst.ca/) for pathway analysis. In this study, the pathway impact value >0.1 was considered to be a metabolic pathway related to the antidepressant effect of drugs, and seven metabolic pathways related to depression were found, namely, D-glutamine and D-glutamate metabolism; phenylalanine, tyrosine, and tryptophan biosynthesis; alanine, aspartic acid, and glutamate metabolism; phenylalanine metabolism; purine metabolism; arginine biosynthesis; and taurine and hypotaurine metabolism (Figure 5A). Interestingly, among these seven metabolic pathways affected by CUMS, single administration of Baishao did not significantly affect any pathway (Figure 5C). However, four pathways were regulated by the single administrated group of Chaihu or the compatibility group (Figures 5B, D). Surprisingly, in these pathways, only the regulations of phenylalanine, tyrosine, and tryptophan biosynthesis and purine metabolism were significantly enhanced after compatibility. Nevertheless, the regulation of purine metabolism by CBHP was more significant, and the number of differential metabolites regulated by CBHP was also greater in purine metabolism (Figure 5E). Phenylalanine metabolism and taurine and hypotaurine metabolism were also regulated after the compatibility of Chaihu and Baishao, whereas the degree of influence was less than that of purine metabolism (Figure 5E). As discussed above, purine metabolism may be a crucial metabolic pathway for the compatibility mechanism of Chaihu and Baishao to play an antidepressant role. To prove this, a targeted quantitative analysis of purine metabolites in the cortex was performed.

### 3.3 Targeted Quantitative Analysis of Purine Metabolites

#### 3.3.1 Method Validation

The declustering potential (DP) and collision energy voltages (CE) for each metabolite were individually selected and listed in Table 2. In addition, the method validation results are shown in Supplementary Material S1, and the experimental data achieved the methodological requirements of tissue sample analysis, which demonstrated that our method was accurate, repeatable, stable, and sensitive enough for targeted quantitative analysis.

**TABLE 2:** MRM-based mass spectrometry parameters for the detection of purine metabolites.

Compounds	Molecular weight	Q1/Q3	DP (V)	CE (eV)	Ion mode
AMP	347.2	348.1–136.1	40	16	+
IMP	348.2	349.2–137.2	35	8	+
GMP	363.1	364.1–152.1	40	12	+
Adenosine	267.2	268.1–136.1	40	20	+
Inosine	268.2	269.1–137.1	21	20	+
Xanthosine	284.23	285.04–152.03	35	15	+
Guanosine	283.24	284.1–152.1	40	20	+
Adenine	135.1	136.0–119.0	36	20	+
Hypoxanthine	136.1	137.0–110.0	36	20	+
Xanthine	152.1	153.1–110.1	35	15	+
Guanine	151.1	152.0–110.0	46	10	+
IS1	301.69	302.1–170.0	35	12	+
IS2	287.359	288.0–213.0	20	20	+

#### 3.3.2 Quantitative Analysis of Targeted Metabolites

**FIGURE 6:**
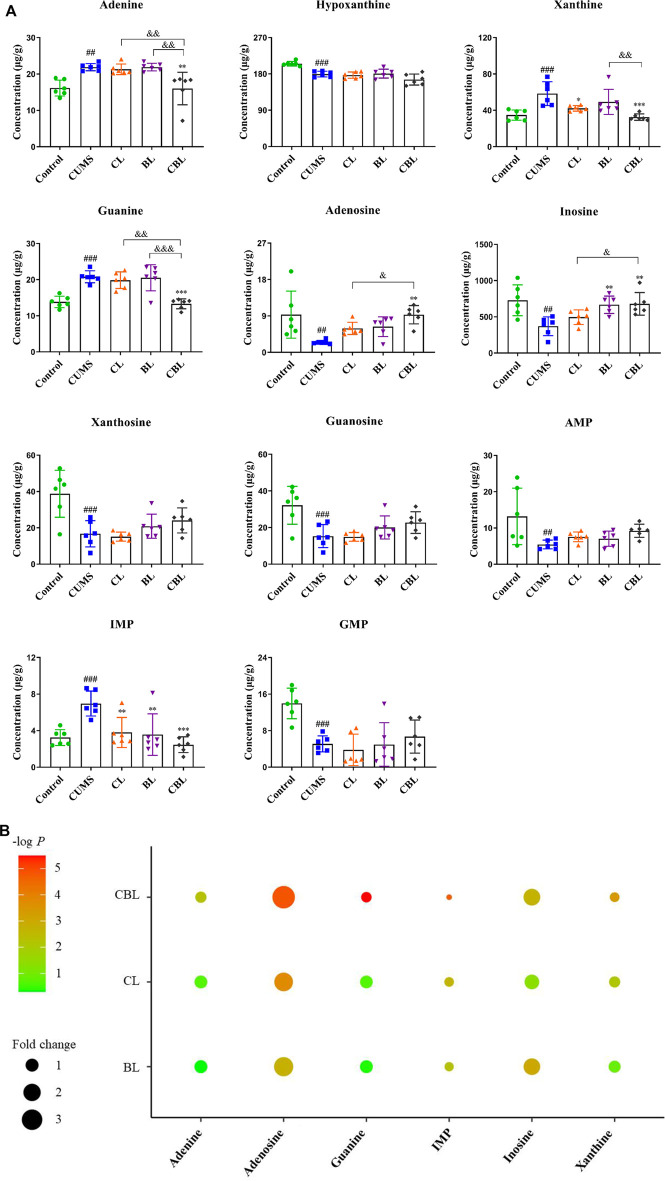
**(A)** Concentration (μg/g) of 11 purine metabolites in the rat cortex. Data are presented as mean ± SD, $\bar{x}$ ± s, *n* = 6. Compared with the control group: ^#^ *p* < 0.05, ^##^ *p* < 0.01, and ^###^ *p* < 0.001; compared with the CUMS group: ^*^ *p* < 0.05, ^**^ *p* < 0.01, and ^***^ *p* < 0.001; compared with the CBHP (CBL) group: ^&^ *p* < 0.05, ^&&^ *p* < 0.01, and ^&&&^ *p* < 0.001. **(B)** Enrichment analysis of different purine metabolites. -log *P*: the significance of different groups compared with the CUMS group; Fold change: relative to the mean of the CUMS group.

The established method was used to measure the levels of purine metabolites in five groups, and the results are shown in Figure 6A. Compared with the control group, the levels of 11 purine metabolites were significantly changed in the CUMS group. Among them, the levels of IMP, adenine, xanthine, and guanine were increased, and the levels of AMP, guanosine monophosphate (GMP), adenosine, inosine, xanthosine, guanosine, and hypoxanthine were decreased. Compared with the CUMS group, CBHP regulated more purine metabolites (six) than individual drugs (two and two); meanwhile, the regulatory effect of CBHP was significantly better than that of Chaihu or Baishao on the five purine metabolites (adenine, xanthine, guanine, adenosine, and inosine), as shown in Figure 6B. Of the five substances, there were two nucleosides (adenosine and inosine) and three bases (adenine, xanthine, and guanine), and the levels of nucleosides were significantly decreased, whereas the levels of bases were significantly increased in the CUMS group. In addition, nucleosides are important raw materials for base synthesis, indicating that the synergistic antidepressant effect of CBHP may be related to the inhibition of base synthesis from nucleosides in purine metabolism. Among the three abnormally elevated bases, excessive accumulation of xanthine was thought to be closely related to mood disorders (Fan et al., 2019), but xanthine synthesis was synergistically inhibited by CBHP, implying that the inhibition of xanthine synthesis played an important role in the synergistic antidepressant effect of CBHP. Subsequently, the content of xanthine and the activities of crucial synthase (XDH and XOD) in the cortex were verified by ELISA kits. Furthermore, previous study has found that excessive synthesis of xanthine was accompanied by the release of ROS (Tanaka et al., 2021). In order to investigate the regulatory effect of CBHP on oxidative stress mediated by ROS, the level of MDA was measured in the cortex. The NLRP3 inflammasome pathway associated with xanthine synthesis was also subsequently measured to further analyze the regulatory effect of CBHP on related signaling pathways.

### 3.4 The Effect of Single Herbs and Herb Pair on Purine Metabolism/Inflammation Signal Pathway

#### 3.4.1 Impacts on Xanthine Synthesis

**FIGURE 7:**
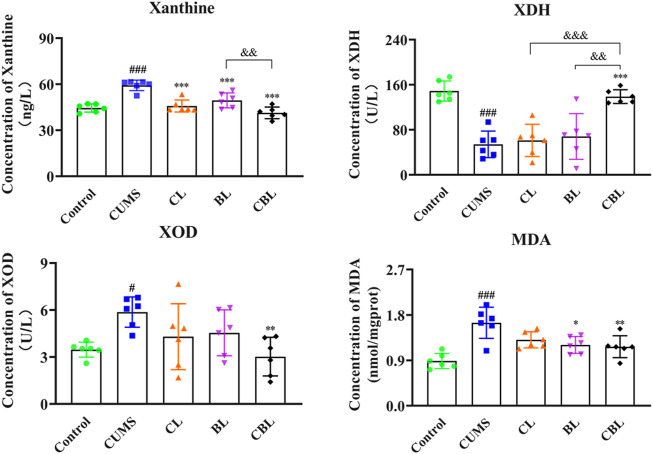
ELISA assay of xanthine related indexes. $\bar{x}$ ± s, *n* = 6. Compared with the control group: ^#^ *p* < 0.05, ^##^ *p* < 0.01, and ^###^ *p* < 0.001; compared with the CUMS group: ^*^ *p* < 0.05, ^**^ *p* < 0.01, and ^***^ *p* < 0.001; compared with the CBHP (CBL) group: ^&^ *p* < 0.05, ^&&^ *p* < 0.01, and ^&&&^ *p* < 0.001.

As the results showed, the levels of xanthine and MDA were significantly reduced by single administration of Chaihu (*p* < 0.001; *p* < 0.05) or Baishao (*p* < 0.001; *p* < 0.05), whereas the activity of XOD and XDH was not significantly affected. After the compatibility of CBHP, not only the contents of xanthine (*p* < 0.001) and MDA (*p* < 0.01) were significantly reduced but also the activity of XOD (*p* < 0.01) was reduced, and the activity of XDH (*p* < 0.001) was increased; of note, the modulatory effects were better than those of the single administered groups (Figure 7).

#### 3.4.2 Inhibitory Effect on the Formation of the NLRP3 Inflammasome

**FIGURE 8:**
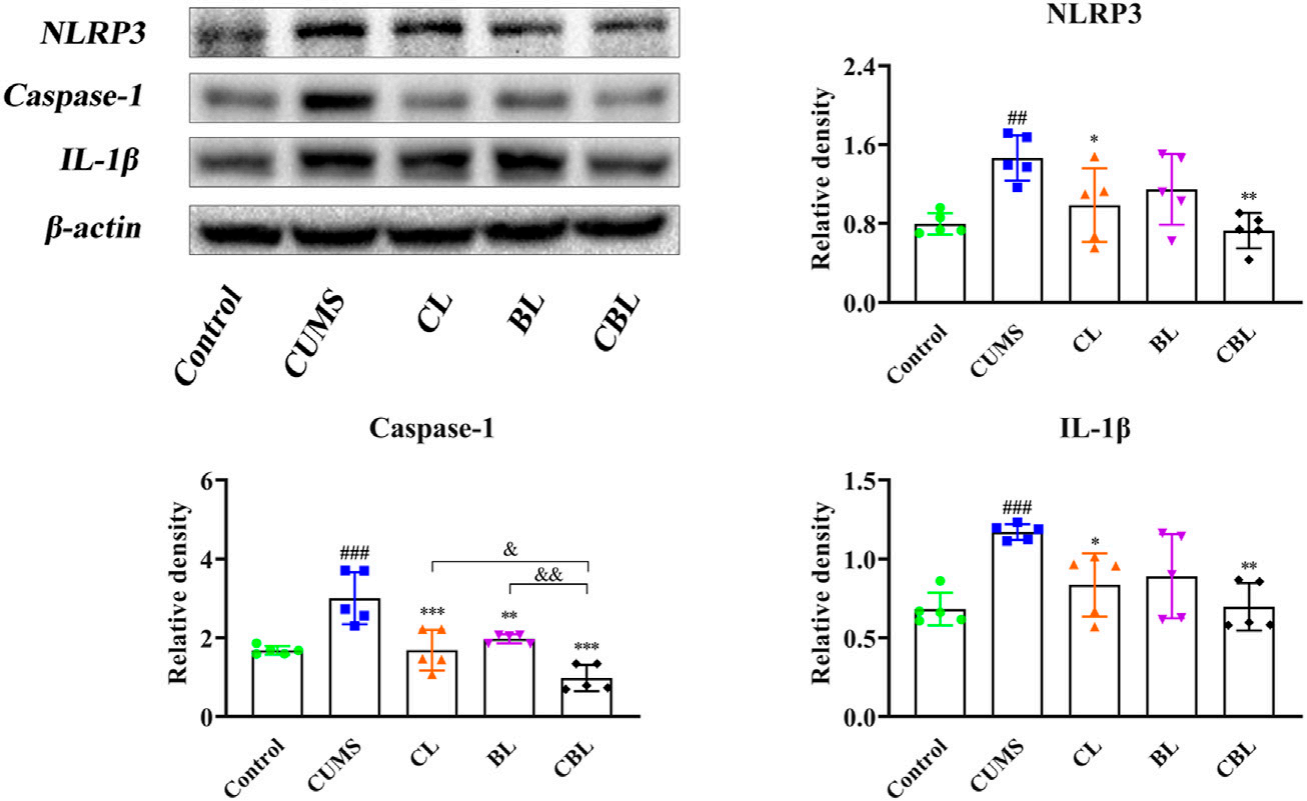
Effects of CBHP on cortical-related proteins of the NLRP3 inflammasome. $\bar{x}$ ± s, *n* = 5. Compared with the control group: ^#^ *p* < 0.05, ^##^ *p* < 0.01, and ^###^ *p* < 0.001; compared with the CUMS group: ^*^ *p* < 0.05, ^**^ *p* < 0.01, and ^***^ *p* < 0.001; compared with the CBHP (CBL) group: ^&^ *p* < 0.05, ^&&^ *p* < 0.01, and ^&&&^ *p* < 0.001.

Compared with the control group, the expression of NLRP3, IL-1*β*, and caspase-1 proteins was increased significantly (*p* < 0.05) in the cortex of CUMS rats, confirming that the occurrence of cortical inflammation was caused by stress. Compared with the CUMS group, the expressions of NLRP3 and IL-1*β* were adjusted by the single administration of Chaihu (*p* < 0.05). However, Baishao had no regulatory effect on the abovementioned two inflammatory proteins, whereas the proteins could be regulated after compatibility (*p* < 0.01), and the significance of CBHP was stronger than that of the single administration of Chaihu*.* Furthermore, CBHP exerted a better-reversed effect than single herbs on caspase-1 (Figure 8).

#### 3.4.3 Resistance to the Release of Inflammatory Factors

**FIGURE 9:**
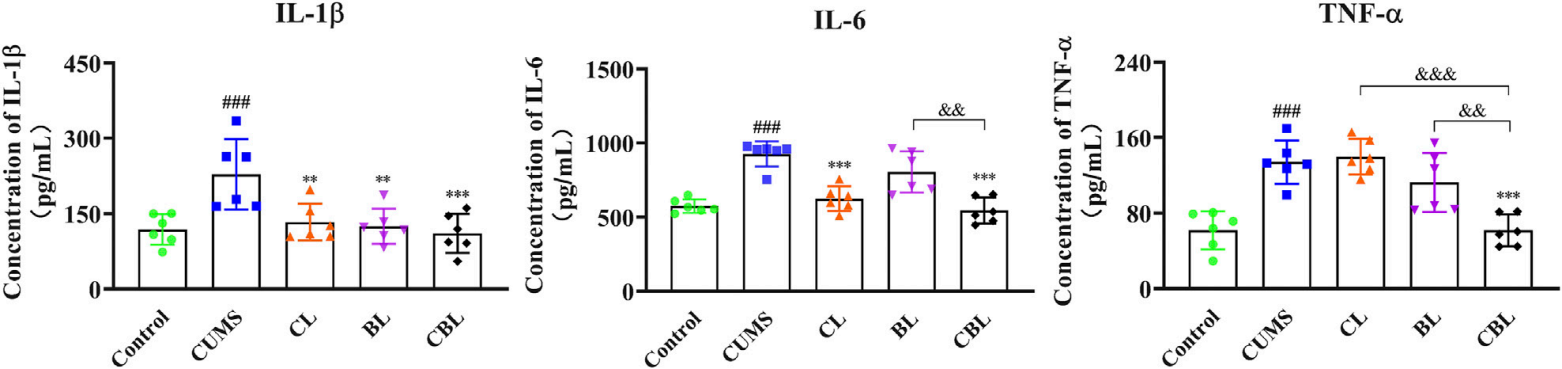
Effects of CBHP on the contents of inflammatory factors. $\bar{x}$ ± s, *n* = 6. Compared with the control group: ^#^ *p* < 0.05, ^##^ *p* < 0.01, and ^###^ *p* < 0.001; compared with the CUMS group: ^*^ *p* < 0.05, ^**^ *p* < 0.01, and ^***^ *p* < 0.001; compared with the CBHP (CBL) group: ^&^ *p* < 0.05, ^&&^ *p* < 0.01, and ^&&&^ *p* < 0.001.

Compared with the control group, the levels of IL-1*β*, IL-6, and TNF-*α* were significantly increased (*p* < 0.001) in the cortex of CUMS rats. According to the results, the levels of IL-1*β* (*p* < 0.01) and IL-6 (*p* < 0.001) were significantly reduced after Chaihu was administrated, and the level of IL-1*β* (*p* < 0.01) was adjusted after the single administration of Baishao. Of interest, as the CBHP was administered, the levels of IL-1*β*, IL-6, and TNF-*α* (*p* < 0.001) were significantly reduced, and the modulatory effects were also significantly improved (Figure 9).

## 4 Discussion

The compatibility mechanism of Chaihu and Baishao in the synergistic treatment of depression was discovered by non-targeted metabolomics combined with targeted quantitative analysis and molecular biology techniques. The results of non-targeted metabolomics indicated that purine metabolism was most related to the synergistic antidepressant effect of CBHP, and the herb pair administration regulated more purine metabolites than single herbs. Subsequently, the synergistic regulation of CBHP on purine metabolites was confirmed by targeted quantitative analysis based on the MRM mode. Consistent with the metabolomics results, CBHP regulated more purine metabolites (six) than single-herb groups (two and two). In addition, compared with non-targeted metabolomics, the results of targeted quantitative analysis for the measurement of purine metabolites were more accurate.

Preclinical studies have reported that the contents of guanosine, inosine, and hypoxanthine decreased, while the contents of IMP and xanthine increased in the brain of depressive models (Cai et al., 2017; Yunfeng Zhou et al., 2019; He et al., 2020). In terms of clinical studies, the same results have been found in the serum of depressive patients, in addition, the level of adenosine was also found to be reduced (Ali-Sisto et al., 2016; Xinyu Zhou et al., 2019). Strikingly, our results were consistent with these reports, and it is found that the improvement effects of CBHP were significantly better than those of single herbs on adenosine, inosine, adenine, guanine, and xanthine.

Adenosine deaminase (ADA) and XOD are key enzymes in purine metabolism, responsible for the decomposition of adenosine and the synthesis of xanthine. A previous study has found that the activities of ADA and XOD in the serum of depressive patients were significantly enhanced (Herken et al., 2007). The present study found that adenosine level decreased, xanthine content increased, and XOD activity enhanced in the cortex of CUMS rats, and our results were consistent with the abovementioned report.

In another study, XOD was found to be a vital component of inflammatory occurrence in depression (Maes et al., 2011). Subsequent studies found that the role of XOD in inducing inflammation was related to release of ROS (Xu et al., 2016; Furuhashi, 2020; Martorell et al., 2021). XDH would be converted to XOD during the production of xanthine from hypoxanthine under pathological conditions, further resulting in the production of ROS, while ROS was a trigger for the activation of the NLRP3 inflammasome. In recent years, numerous studies have reported the release of ROS in the cortex of the CUMS model (Zhang et al., 2016; Wan et al., 2017; Zou et al., 2020). Consistently, we conducted a quantitative analysis of XOD and XDH, and the results indicated that the activities of the two enzymes showed a reverse trend after stress. In addition, the level of MDA increased, indicating that oxidative stress occurred in the cortex after the conversion of XDH to XOD. From this perspective, CBHP had stronger inhibitory effects than single herbs on the conversion of XDH and the production of MDA. Furthermore, the expression levels of the NLRP3 inflammasome and the release of inflammatory factors were significantly increased in the CUMS group. Compared with single herbs, these oxidative stress and inflammatory responses were better modulated after CBHP administration.

**FIGURE 10:**
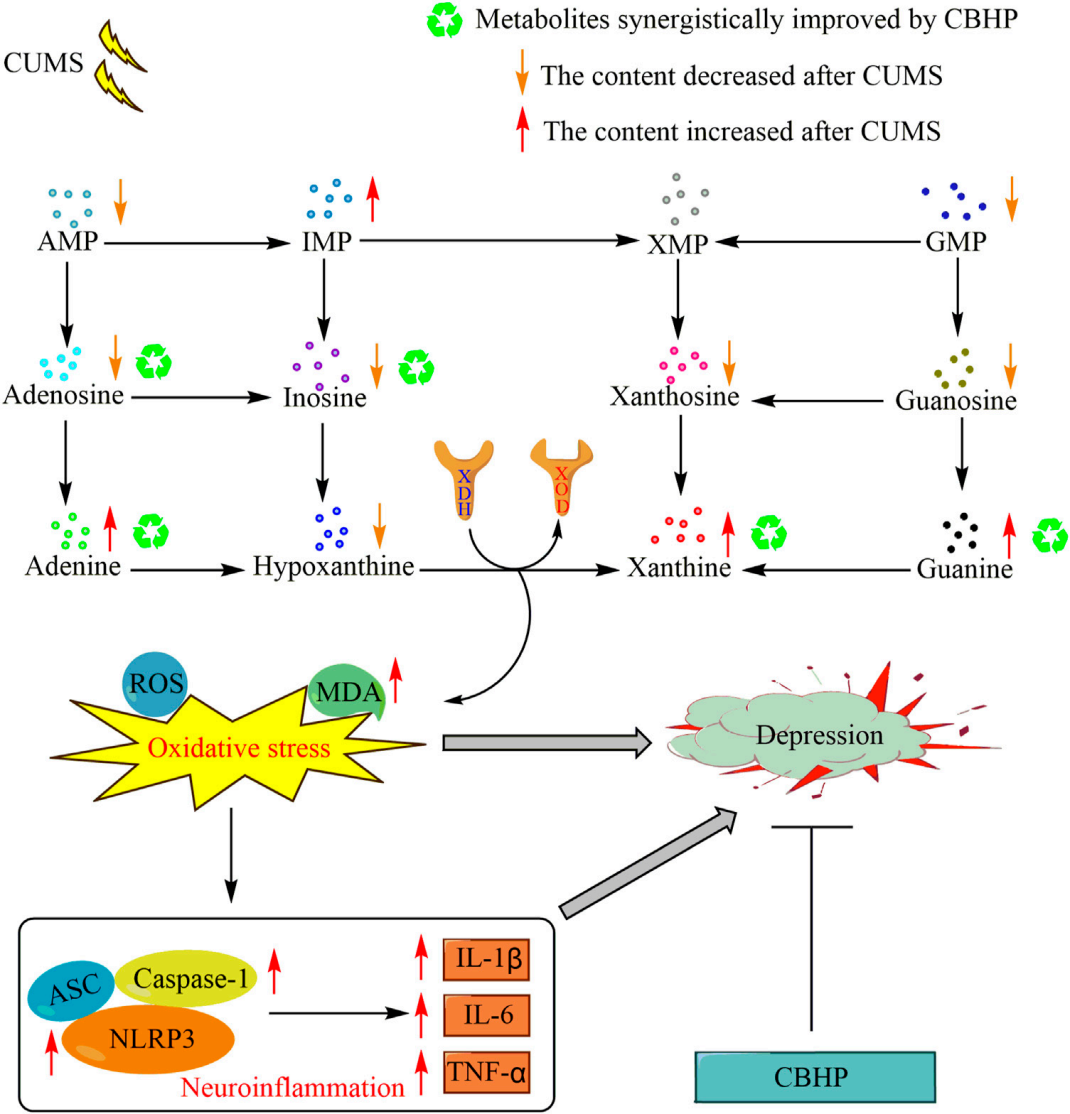
Potential compatibility mechanism of Chaihu and Baishao for exerting a synergistic antidepressant effect.

Collectively, this study elucidated a novel mechanism in the pathogenesis of depression, and the results showed that the occurrence of oxidative stress and the activation of the NLRP3 inflammasome pathway were potentially mediated by excessive synthesis of xanthine, which in turn induced depression. Compared with single herbs, these biological changes were improved more strongly by CBHP, indicating that the synergistic antidepressant effect of CBHP was related to the inhibition of this pathway. The potential compatibility mechanism of Chaihu and Baishao for exerting a synergistic antidepressant effect is shown in Figure 10.

Although the new compatibility mechanism of Chaihu and Baishao for treating depression was reported in this study, the following shortcomings were still observed. Other purine metabolites involved in the compatibility mechanism of Chaihu and Baishao have not been fully detected and are yet to be found. In addition, the changes in ROS levels need to be verified in live animals.

## 5 Conclusion

The present study demonstrated that the regulation of purine metabolism, the suppression of oxidative stress, and inflammatory responses in the cortex were involved in the synergistic antidepressant effect of CBHP.

## Data Availability

The original contributions presented in the study are included in the article/Supplementary Material; further inquiries can be directed to the corresponding author.
