# Supplementary material for: Integration of Non-Targeted Metabolomics and Targeted Quantitative Analysis to Elucidate the Synergistic Antidepressant Effect of Bupleurum Chinense DC-Paeonia Lactiflora Pall Herb Pair by Regulating Purine Metabolism
Source: Front Pharmacol. 2022 Jun 30;13:900459. doi: 10.3389/fphar.2022.900459 (PMC9280301; doi:10.3389/fphar.2022.900459)
Supplement: Supplementary file 1 [file DataSheet1.DOC]

**Table S1** Mobile phase and injection time.

| Time(min) | Solvent A | Solvent B |
| --- | --- | --- |
|  | (0.1% Formic acid water) | (Acetonitrile) |
| 0~2 | 98% A | 2% B |
| 2~3 | 98~65% A | 2~35% B |
| 3~10 | 65%~30% A | 35~70% B |
| 10~12 | 30~15% A | 70~85% B |
| 12~16 | 15%~2% A | 85~98% B |
| 16~18 | 2% A | 98% B |
| 18~20 | 2%~98% A | 98~2% B |
| 20~23 | 98%A | 2% B |

**Table S2** Targeted metabolites and internal standard reference substances.

| No. | Compounds | Specifications | Molecular weight | CAS | Stock solution（mg/mL） | Mixed standard range（μg/mL） |
| --- | --- | --- | --- | --- | --- | --- |
| 1 | AMP | 1000 mg | 347.22 | 61-19-8 | 4.17 | 0.3125-80 |
| 2 | IMP | 5 mg | 348.21 | 131-99-7 | 1.00 | 0.3125-80 |
| 3 | GMP | 500 mg | 407.18 | 5550-12-9 | 7.00 | 0.46875-120 |
| 4 | Adenine | 500 mg | 135.13 | 73-24-5 | 4.30 | 0.3125-80 |
| 5 | Hypoxanthine | 10 mg | 136.11 | 68-94-0 | 1.00 | 0.78125-200 |
| 6 | Xanthine | 100 mg | 152.11 | 69-89-6 | 4 .00 | 0.3125-80 |
| 7 | Guanine | 500 mg | 151.13 | 73-40-5 | 2.16 | 0.3125-80 |
| 8 | Adenosine | 500 mg | 267.25 | 58-61-7 | 5.76 | 0.3125-80 |
| 9 | Inosine | 500 mg | 268.23 | 58-63-9 | 20.53 | 3.125-800 |
| 10 | Xanthosine | 5 mg | 284.23 | 146-80-5 | 2.24 | 0.3125-80 |
| 11 | Guanosine | 500 mg | 283.24 | 118-00-3 | 6.16 | 0.3125-80 |
| 12 | IS1 | 5 mg | 301.69 | 146-77-0 | 1.00 | 1.2 |
| 13 | IS2 | 5 mg | 287.35 | 357-70-0 | 1.00 | 0.15 |

**Table S3** Preparation of quality control solution.

| Compounds | QC1 | QC2 | QC3 | LLOQ |
| --- | --- | --- | --- | --- |
| AMP, IMP, Adenine, Xanthine, Guanine, Adenosine, Xanthine, Guanosine | 40 μg/mL | 5 μg/mL | 0.625 μg/mL | 0.3125  μg/mL |
| GMP | 60 μg/mL | 7.5 μg/mL | 0.9375 μg/mL | 0.46875 μg/mL |
| Hypoxanthine | 100 μg/mL | 12.5 μg/mL | 1.5625 μg/mL | 0.78125 μg/mL |
| Inosine | 400 μg/mL | 50 μg/mL | 6.25 μg/mL | 3.125  μg/mL |

**Table S4** The RSD values of the relative peak areas of 20 random ions in the 6 QC samples.

| NO. | m/z | TR  (min) | Relative  peak area | RSD  (%) | Ion mode |
| --- | --- | --- | --- | --- | --- |
| 1 | 148.0419 | 1.017 | 0.0112 | 11.34 | + |
| 2 | 132.1018 | 1.250 | 0.0080 | 11.41 | + |
| 3 | 268.1035 | 1.252 | 0.0021 | 9.03 | + |
| 4 | 269.0876 | 1.258 | 0.0056 | 4.14 | + |
| 5 | 198.0371 | 1.310 | 0.0069 | 13.13 | + |
| 6 | 132.1018 | 2.687 | 0.0024 | 9.64 | + |
| 7 | 149.0596 | 4.595 | 0.0042 | 11.79 | + |
| 8 | 205.097 | 4.907 | 0.0026 | 14.15 | + |
| 9 | 275.2773 | 8.262 | 0.0023 | 5.92 | + |
| 10 | 274.2736 | 8.263 | 0.0147 | 7.00 | + |
| 11 | 362.3259 | 8.460 | 0.0013 | 5.90 | + |
| 12 | 496.3213 | 11.722 | 0.0132 | 10.62 | + |
| 13 | 523.3582 | 12.258 | 0.0012 | 5.60 | + |
| 14 | 149.0232 | 13.095 | 0.0028 | 9.69 | + |
| 15 | 524.3707 | 13.413 | 0.0025 | 8.25 | + |
| 16 | 485.3156 | 14.207 | 0.0036 | 6.24 | + |
| 17 | 352.3024 | 14.548 | 0.0035 | 8.00 | + |
| 18 | 256.2631 | 14.814 | 0.0081 | 11.22 | + |
| 19 | 291.2677 | 15.558 | 0.0010 | 6.12 | + |
| 20 | 141.9832 | 20.587 | 0.0043 | 14.1 | + |


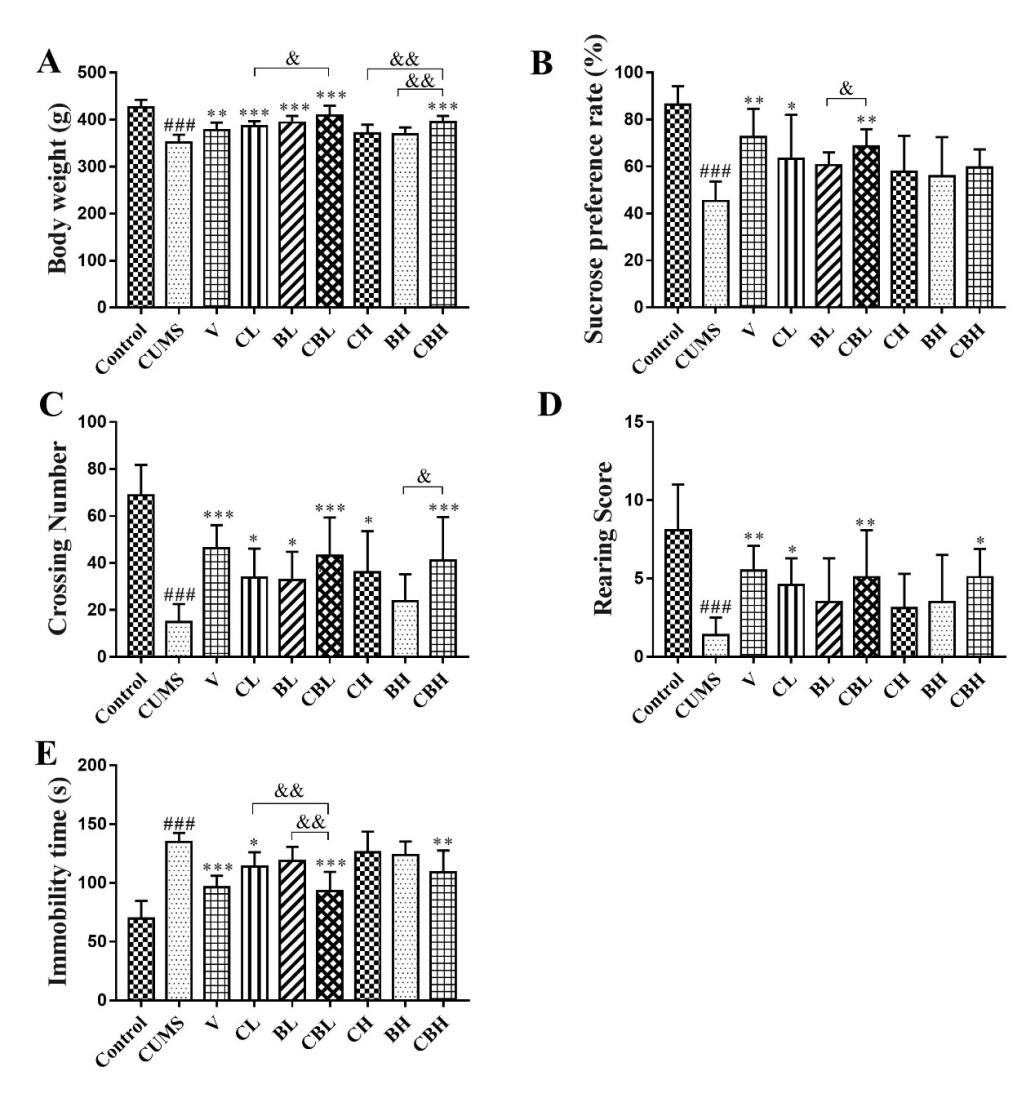


**Figure S1** Comparison of antidepressant effect between Chaihu and Baishao before and after compatibility. **(A)** Body weight. **(B)** Sucrose preference. **(C)** The crossing number in OFT. **(D)** The rearing score in OFT. **(E)** Immobility time in FST. All data were expressed as mean ± SD, n = 9. Compared with control group: ^###^*P*<0.001; compared with CUMS group: ^*^*P*<0.05 ^**^*P*<0.01 ^***^*P*<0.001; compared with Chaihu-Baishao group: ^&^*P*<0.05, ^&&^*P*<0.01, ^&&&^*P*<0.001.


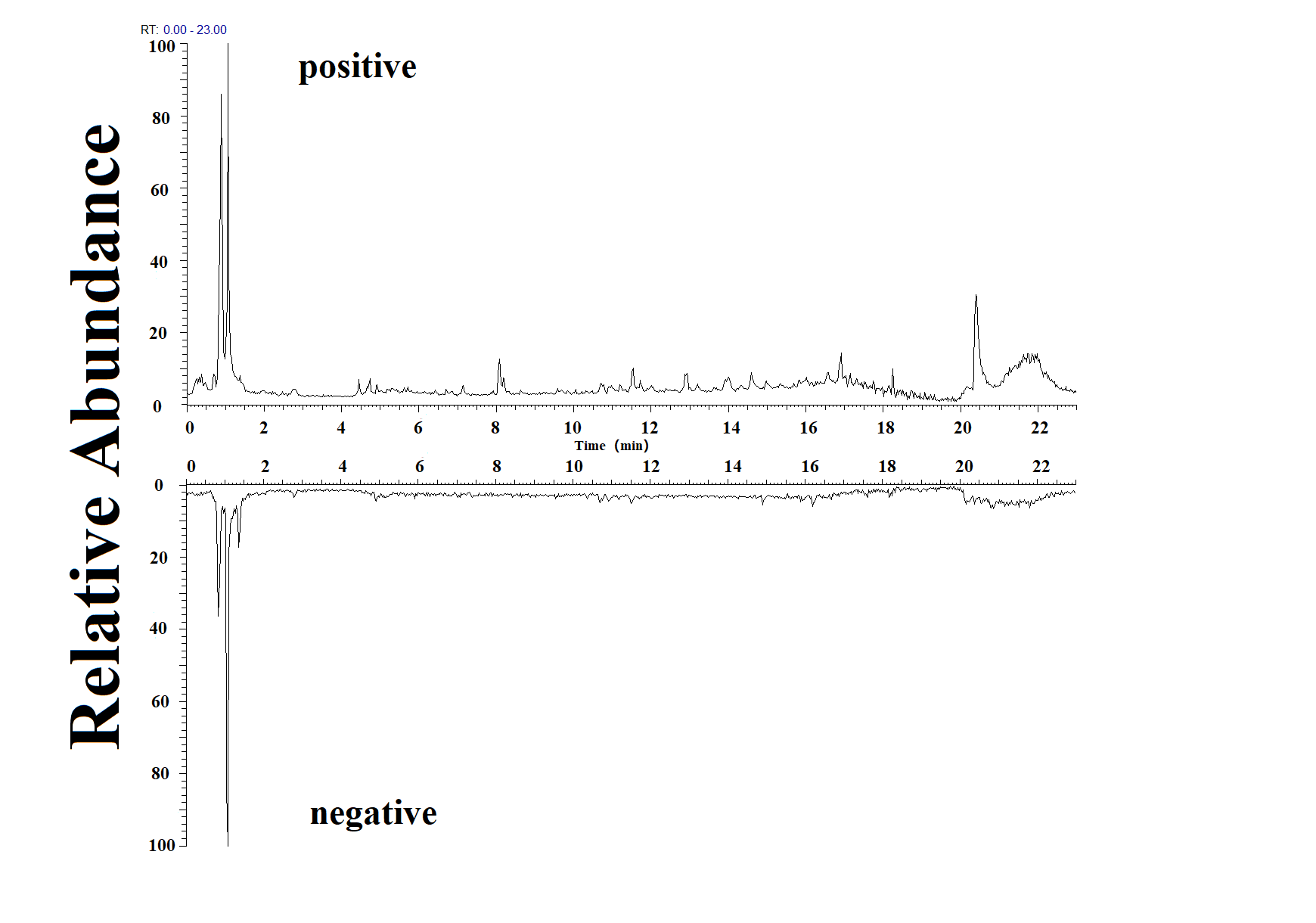


**Figure S2** Typical LC-MS/MS spectrum of control rat cortex.


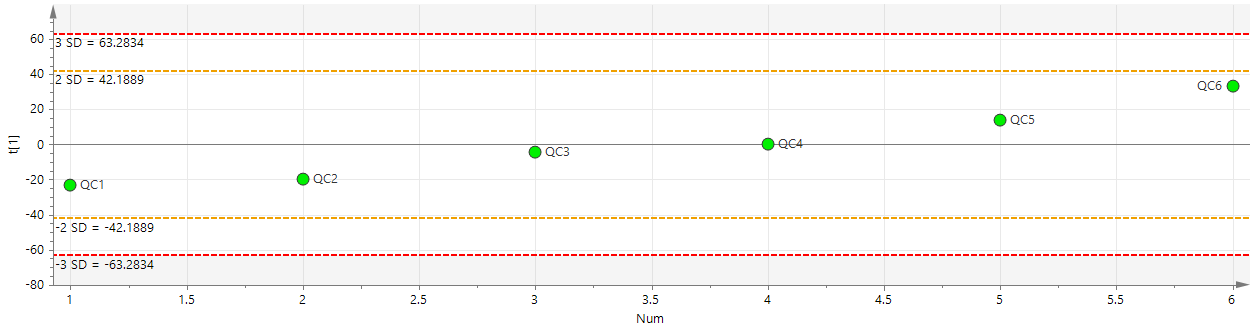


**Figure S3** The deviation of QC samples.

**Methodological validation of targeted quantitative analysis**

**1 Selectivity**

The total ion chromatogram and MRM chromatogram of the blank solution, QC-2 and normal rat cerebral cortex sample were shown in Figure S4.


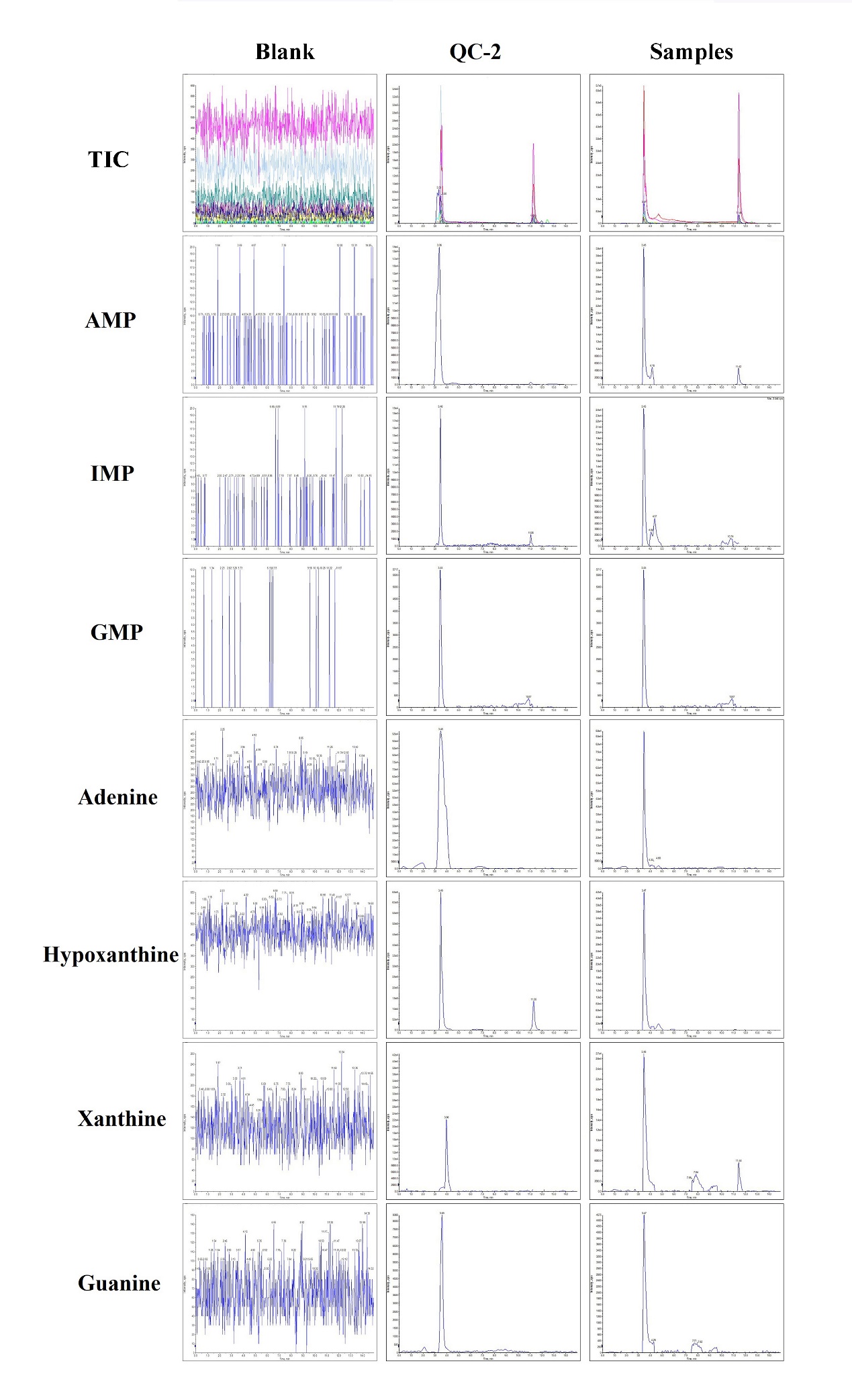


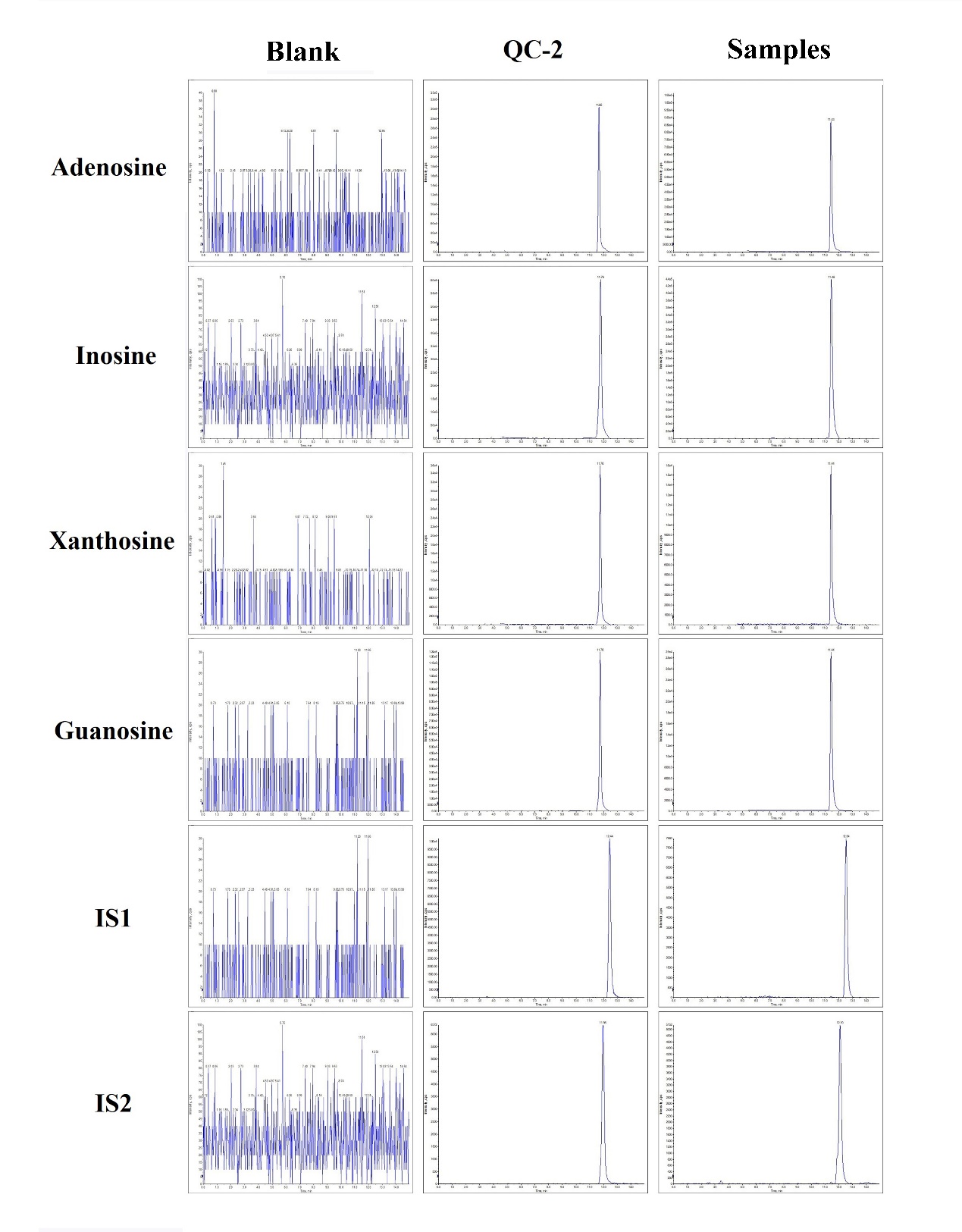


**Figure S4** Selectivity.

**2 Linearity and concentration range**

The regression equation, correlation coefficient (R^2^), linear range and corresponding internal standard were shown in Table S5. As shown in Figure S5, 11 metabolites in purine metabolism have good linearity.

**Table S5** Linearity relationship of 11 metabolites in purine metabolism.

| Metabolites | TR  (min) | Regression equation (n = 9) | R^2^ | Range  (μg/mL) | LLOQ | LOD | IS |
| --- | --- | --- | --- | --- | --- | --- | --- |
| Adenine | 3.34 | y=0.6257x+1.2885 | 0.9998 | 0.3125-80 | 0.3125 | 0.104167 | IS2 |
| Hypoxanthine | 3.53 | y=0.306x+0.2602 | 0.9994 | 0.78125-200 | 0.78125 | 0.260417 | IS2 |
| Xanthine | 3.43 | y=0.1477x+0.1554 | 0.9966 | 0.3125-80 | 0.3125 | 0.104167 | IS2 |
| Guanine | 3.38 | y=0.0561x+0.08 | 1.0000 | 0.3125-80 | 0.3125 | 0.104167 | IS2 |
| Adenosine | 11.18 | y=2.27x-0.1066 | 0.9997 | 0.3125-80 | 0.3125 | 0.104167 | IS1 |
| Inosine | 11.28 | y=0.272x+3.4985 | 0.9978 | 3.125-800 | 3.125 | 1.041667 | IS1 |
| Xanthosine | 11.26 | y=0.145x+0.0559 | 0.9996 | 0.3125-80 | 0.3125 | 0.104167 | IS1 |
| Guanosine | 11.26 | y=0.3931x+0.045 | 0.9998 | 0.3125-80 | 0.3125 | 0.104167 | IS1 |
| AMP | 3.32 | y=0.154x-0.094 | 0.9998 | 0.3125-80 | 0.3125 | 0.104167 | IS2 |
| IMP | 3.43 | y=0.0393x+0.0167 | 0.9996 | 0.3125-80 | 0.3125 | 0.104167 | IS2 |
| GMP | 3.41 | y=0.0291x+0.0456 | 0.9998 | 0.46875-120 | 0.46875 | 0.15625 | IS2 |

**
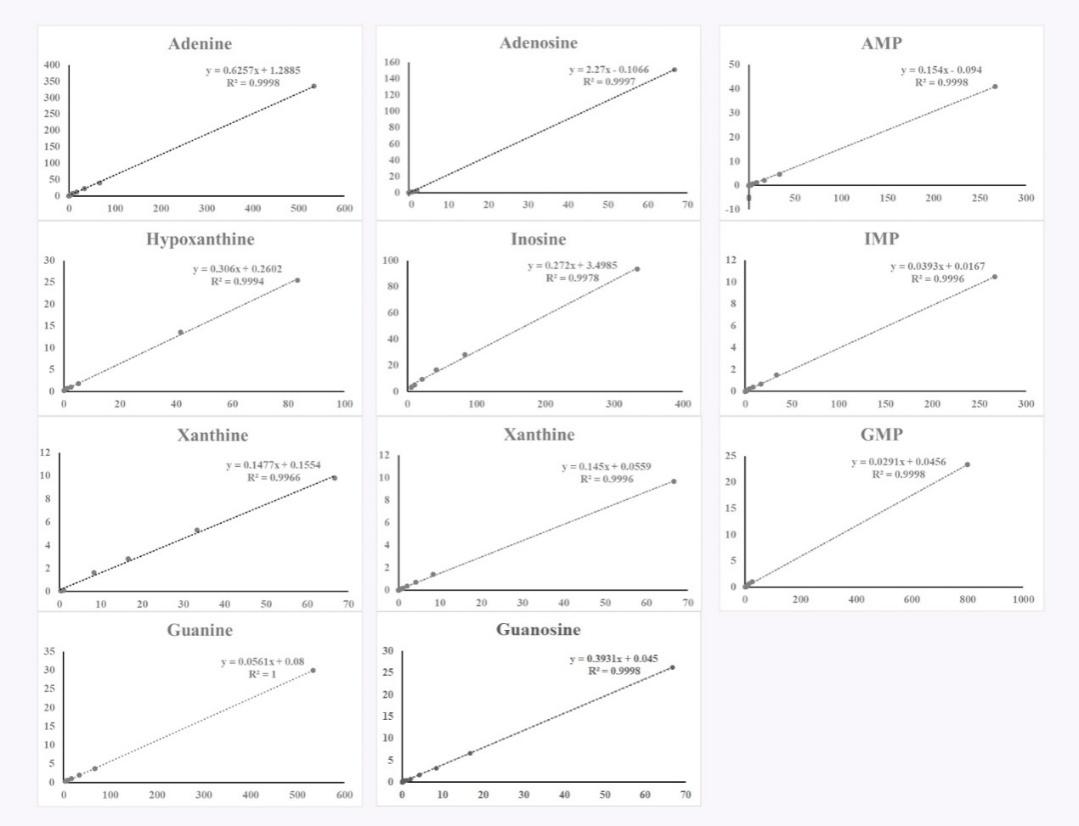
**

**Figure S5** Standard curves of 11 metabolites in purine metabolism.

**3 Precision**

As shown in Table S6, the precision deviation met the requirement under the established method, indicating that the established method was suitable for the detection of samples.

**Table S6** The RSD values of the intra-day and inter-day precision of 11 metabolites.

| Metabolites | Theoretical concentration (μg/mL) | Intra-day (n = 6) | | Inter-day (n = 6) | |
| --- | --- | --- | --- | --- | --- |
|  |  | Measured (μg/mL) | RSD (%) | Measured  (μg/mL) | RSD (%) |
| Adenine | 40 | 37.41±1.687 | 4.45 | 38.91±3.008 | 4.49 |
|  | 5 | 4.931±0.047 | 1.96 | 4.888±0.110 | 2.24 |
|  | 0.625 | 0.5572±0.024 | 5.62 | 0.5654±0.031 | 5.55 |
|  | 0.3125 | 0.309±0.009 | 2.36 | 0.3132±0.009 | 2.99 |
| Hypoxanthine | 100 | 88.94±3.951 | 4.69 | 89.6±3.984 | 4.45 |
|  | 12.5 | 12.3±0.491 | 3.12 | 12.09±0.507 | 4.20 |
|  | 1.5625 | 1.194±0.041 | 3.65 | 1.23±0.051 | 4.12 |
|  | 0.78125 | 0.7895±0.015 | 3.25 | 0.7845±0.025 | 3.23 |
| Xanthine | 40 | 31.72±1.753 | 4.45 | 31.67±1.421 | 4.49 |
|  | 5 | 4.482±0.103 | 3.58 | 4.464±0.172 | 3.84 |
|  | 0.625 | 0.5872±0.015 | 3.78 | 0.606±0.026 | 4.33 |
|  | 0.3125 | 0.2275±0.009 | 5.59 | 0.2171±0.025 | 11.43 |
| Guanine | 40 | 38.53±2.453 | 6.05 | 39.23±2.326 | 5.93 |
|  | 5 | 4.695±0.308 | 5.19 | 4.752±0.341 | 7.18 |
|  | 0.625 | 0.6426±0.019 | 5.02 | 0.6411±0.032 | 4.98 |
|  | 0.3125 | 0.2958±0.011 | 4.18 | 0.2987±0.014 | 4.53 |
| Adenosine | 40 | 39.07±1.531 | 4.59 | 38.84±1.731 | 4.46 |
|  | 5 | 4.731±0.146 | 4.17 | 5.088±0.343 | 6.73 |
|  | 0.625 | 0.5369±0.029 | 3.41 | 0.5336±0.020 | 3.83 |
|  | 0.3125 | 0.3088±0.005 | 1.58 | 0.3071±0.005 | 1.65 |
| Inosine | 400 | 412.1±17.130 | 4.91 | 406.2±20.210 | 4.98 |
|  | 50 | 43.23±2.862 | 5.68 | 44.17±2.677 | 6.06 |
|  | 6.25 | 5.497±0.359 | 5.72 | 5.523±0.307 | 5.55 |
|  | 3.125 | 3.349±0.249 | 8.94 | 3.482±0.339 | 9.72 |
| Xanthosine | 40 | 39.6±1.618 | 4.80 | 39.31±1.858 | 4.73 |
|  | 5 | 5.22±0.364 | 5.25 | 5.465±0.359 | 6.57 |
|  | 0.625 | 0.4058±0.042 | 9.08 | 0.3778±0.042 | 11.22 |
|  | 0.3125 | 0.3418±0.015 | 11.22 | 0.3198±0.039 | 15.76 |
| Guanosine | 40 | 38.46±1.555 | 4.30 | 38.01±1.574 | 4.14 |
|  | 5 | 4.176±0.147 | 4.37 | 4.4±0.252 | 5.74 |
|  | 0.625 | 0.6068±0.062 | 9.16 | 0.597±0.053 | 8.97 |
|  | 0.3125 | 0.3192±0.017 | 4.83 | 0.3107±0.016 | 5.12 |
| AMP | 40 | 34.75±1.582 | 4.79 | 34.72±1.675 | 4.82 |
|  | 5 | 5.038±0.114 | 2.27 | 4.936±0.142 | 2.87 |
|  | 0.625 | 0.5938±0.016 | 2.87 | 0.6021±0.017 | 2.89 |
|  | 0.3125 | 0.3291±0.048 | 6.57 | 0.3234±0.036 | 11.26 |
| IMP | 40 | 38.9±1.700 | 4.77 | 38.6±1.793 | 4.65 |
|  | 5 | 5.144±0.332 | 4.18 | 5.034±0.236 | 4.68 |
|  | 0.625 | 0.5939±0.014 | 3.26 | 0.6012±0.020 | 3.26 |
|  | 0.3125 | 0.3133±0.012 | 5.03 | 0.3033±0.024 | 8.02 |
| GMP | 60 | 58.43±2.995 | 4.59 | 58.11±3.203 | 5.51 |
|  | 7.5 | 7.393±0.071 | 1.83 | 7.431±0.144 | 1.94 |
|  | 0.9375 | 1.219±0.127 | 6.73 | 1.366±0.139 | 10.15 |
|  | 0.46875 | 0.3708±0.017 | 7.60 | 0.3281±0.062 | 18.82 |

**4 Stability**

As shown in Table S7, the RSD values of two different treatments met the requirement, indicating that the established method was stable for the detection of the samples.

**Table S7** Stability of 11 metabolites in purine metabolism.

| Metabolites  (n = 3) | Theoretical concentration (μg/mL) | Repeated freeze-thaw 3 times at -20 °C | | Placed in an autosampler at 4 °C for 24 hours | |
| --- | --- | --- | --- | --- | --- |
|  |  | Measured (μg/mL) | RSD  (%) | Measured (μg/mL) | RSD (%) |
| Adenine | 40 | 38.64±2.034 | 5.26 | 38.88±2.454 | 6.31 |
|  | 0.625 | 0.5489±0.020 | 3.58 | 0.5458±0.030 | 5.44 |
| Hypoxanthine | 100 | 87.55±2.234 | 2.55 | 88.78±3.425 | 3.86 |
|  | 1.5625 | 1.213±0.031 | 2.52 | 1.215±0.035 | 2.92 |
| Xanthine | 40 | 31.75±0.402 | 1.27 | 31.62±0.869 | 2.75 |
|  | 0.625 | 0.5913±0.015 | 2.47 | 0.6002±0.029 | 4.73 |
| Guanine | 40 | 38.66±1.579 | 4.08 | 39.03±2.337 | 5.99 |
|  | 0.625 | 0.6261±0.039 | 6.24 | 4.16±0.244 | 5.85 |
| Adenosine | 40 | 39.08±1.210 | 3.10 | 40.1±1.012 | 2.52 |
|  | 0.625 | 0.5204±0.016 | 3.12 | 0.5326±0.020 | 3.79 |
| Inosine | 400 | 397.5±8.775 | 2.21 | 412.4±18.970 | 4.60 |
|  | 6.25 | 5.371±0.128 | 2.38 | 5.409±0.196 | 3.62 |
| Xanthosine | 40 | 39.42±0.620 | 1.57 | 40.2±1.325 | 3.30 |
|  | 0.625 | 0.353±0.011 | 3.02 | 0.3546±0.012 | 3.27 |
| Guanosine | 40 | 38.77±0.719 | 1.85 | 38.95±0.914 | 2.35 |
|  | 0.625 | 0.5966±0.032 | 5.29 | 0.5959±0.031 | 5.19 |
| AMP | 40 | 35.2±1.804 | 5.12 | 34.38±1.890 | 5.50 |
|  | 0.625 | 0.5982±0.016 | 2.67 | 0.5978±0.021 | 3.54 |
| IMP | 40 | 39.28±0.856 | 2.18 | 38.38±0.991 | 2.58 |
|  | 0.625 | 0.618±0.005 | 0.88 | 0.6056±0.024 | 3.89 |
| GMP | 60 | 59.66±1.386 | 2.32 | 57.93±3.193 | 5.51 |
|  | 0.9375 | 1.435±0.012 | 0.84 | 1.415±0.037 | 2.64 |

**5 Extraction recovery rate and matrix effect**

As shown in Table S8, the RSD values of extraction recovery rate ranged from 0.69% to 3.66%, and the RSD range of matrix effect was from 1.29% to 4.77%, indicating that the established method was stable and reliable in extraction recovery rate and matrix effect.

**Table S8** Extraction recovery rate and matrix effect of 11 metabolites in purine metabolism.

| Metabolites | Theoretical concentration (μg/mL) | Extraction recovery rate  (n = 6) | | Matrix effect  (n = 6) | |
| --- | --- | --- | --- | --- | --- |
|  |  | Mean±SD (%) | RSD (%) | Mean±SD (%) | RSD (%) |
| Adenine | 40 | 94.98±3.063 | 3.23 | 103.6±4.937 | 4.77 |
|  | 0.625 | 97.69±1.447 | 1.48 | 100.9±1.305 | 1.29 |
| Hypoxanthine | 100 | 97.75±1.985 | 2.03 | 102.4±3.629 | 3.54 |
|  | 1.5625 | 98.37±1.792 | 1.82 | 99.09±4.082 | 4.12 |
| Xanthine | 40 | 97.09±2.248 | 2.32 | 102.6±2.480 | 2.42 |
|  | 0.625 | 97.46±1.508 | 1.55 | 99.93±2.775 | 2.78 |
| Guanine | 40 | 96.81±2.223 | 2.30 | 101.6±4.196 | 4.13 |
|  | 0.625 | 95.69±2.499 | 2.61 | 100.2±3.150 | 3.14 |
| Adenosine | 40 | 97.13±3.028 | 3.12 | 98.76±2.697 | 2.74 |
|  | 0.625 | 98.77±0.857 | 0.87 | 97.08±2.721 | 2.80 |
| Inosine | 400 | 99.5±0.6883 | 0.69 | 99.72±1.978 | 1.98 |
|  | 6.25 | 96.28±3.014 | 3.13 | 98.09±2.059 | 2.10 |
| Xanthosine | 40 | 95.26±2.614 | 2.74 | 98.79±3.421 | 3.46 |
|  | 0.625 | 96.5±2.071 | 2.15 | 98.1±2.578 | 2.63 |
| Guanosine | 40 | 98.39±1.603 | 1.63 | 100±2.483 | 2.48 |
|  | 0.625 | 94.22±1.729 | 1.84 | 97.31±2.637 | 2.71 |
| AMP | 40 | 95.58±3.499 | 3.66 | 104±4.367 | 4.20 |
|  | 0.625 | 97.74±1.826 | 1.86 | 100.2±2.569 | 2.56 |
| IMP | 40 | 97.43±1.406 | 1.44 | 101.4±4.078 | 4.02 |
|  | 0.625 | 97.75±1.413 | 1.45 | 100.9±1.428 | 1.42 |
| GMP | 60 | 97.49±1.649 | 1.69 | 101.6±3.483 | 3.43 |
|  | 0.9375 | 95.06±2.960 | 3.11 | 100.2±2.351 | 2.35 |
| IS1 | 1.2 (QC1) | 96.95±1.998 | 2.06 | **-** | **-** |
|  | 1.2 (QC3) | 97.66±1.835 | 1.88 | **-** | **-** |
| IS2 | 0.15 (QC1) | 96.99±1.938 | 2.00 | **-** | **-** |
|  | 0.15 (QC3) | 97.63±1.943 | 1.99 | **-** | **-** |

**6 Dilution effect**

As shown in Table S9, the RSD values ranged from 0.05% to 1.07%, indicating that the method was stable and reliable in terms of dilution effect.

**Table S9** Dilution effect of 11 metabolites in purine metabolism.

| Metabolites | 128 times dilution (n = 5) | |
| --- | --- | --- |
|  | Measured (μg/mL) | RSD (%) |
| Adenine | 0.6189±0.002 | 0.40 |
| Hypoxanthine | 1.552±0.005 | 0.30 |
| Xanthine | 0.6049±0.003 | 0.46 |
| Guanine | 0.6147±0.004 | 0.58 |
| Adenosine | 0.6084±0.004 | 0.73 |
| Inosine | 6.212±0.024 | 0.39 |
| Xanthosine | 0.607±0.005 | 0.76 |
| Guanosine | 0.6156±0.007 | 1.07 |
| AMP | 0.6028±0.001 | 0.20 |
| IMP | 0.6171±0.001 | 0.05 |
| GMP | 0.9047±0.002 | 0.18 |
